# Metal‐Ligand Complexes as Multifunctional Platforms for Photo‐Activated Cancer Therapy

**DOI:** 10.1002/advs.202504575

**Published:** 2025-08-11

**Authors:** Jeonga Kim, Saehan Choi, Yoonsung Nam

**Affiliations:** ^1^ Department of Materials Science and Engineering Korea Advanced Institute of Science and Technology 291 Daehak‐ro, Yuseong‐gu Daejeon 34141 Republic of Korea; ^2^ Department of Biological Sciences Korea Advanced Institute of Science and Technology 291 Daehak‐ro Yuseong‐gu Daejeon 34141 Republic of Korea

**Keywords:** metal‐ligand complex, photodynamic therapy, photosensitizer, photothermal therapy

## Abstract

Metal‐ligand complexes have emerged as versatile platforms for photo‐activated therapy (PAT) for cancer, including photothermal therapy, photodynamic therapy, and photo‐activated chemotherapy. This review explores the unique optical properties and tunable characteristics of these complexes, which enable effective reactive oxygen species generation and near‐infrared light absorption for targeted cancer cell destruction. Metal‐ligand complexes are classified based on their ligands and metal ions. The ligands include tetrapyrrolic structures, polypyridine, and polyphenols, while the metal ions comprise transition and noble metals. Their individual contributions to enhancing therapeutic efficacy are also explored. Fundamental interactions, including ligand‐to‐metal charge transfer and metal‐ligand coordination, are examined. These interactions significantly influence the photoreactivity and biocompatibility of the complexes. Recent advances integrating metal‐ligand complexes with other modalities, such as chemo‐phototherapy and immunotherapy, are highlighted. These combinations offer promising approaches for synergistic tumor eradication. The challenges of ligand diversity, delivery optimization, and the underexplored mechanisms in PAT are also discussed. By integrating recent progress and prospective applications, this review emphasizes the critical importance of metal‐ligand complexes in innovating cancer theranostic technologies.

## Graphical abstract

Metal‐ligand complexes serve as multifunctional platforms for photo‐activated cancer therapy, facilitating photothermal and photodynamic effects through light absorption and reactive oxygen species generation. This review categorizes these complexes by ligand and metal types, examines their roles in combined therapeutic approaches, and explores strategies to enhance their efficacy. Future directions and mechanistic insights for advancing their clinical potential are also discussed.

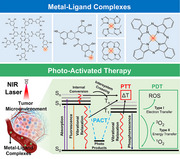

## 1 Introduction

Photo‐activated therapy (PAT) has emerged as a key modality in current cancer treatment, offering a targeted and minimally invasive approach that complements traditional therapies such as surgery, chemotherapy, and radiotherapy. PAT includes photothermal therapy (PTT), photodynamic therapy (PDT), and photo‐activated chemotherapy (PACT). These approaches use specific wavelengths of light to generate localized hyperthermia or to activate therapeutic agents, enabling the selective destruction of cancer cells while minimizing damage to surrounding healthy tissues. PAT is required for effective cancer treatment because it can address critical challenges through unique advantages. First, PAT allows for precise localization of treatment, as the activation of photosensitizers occurs only in the presence of specific light exposure. This precision minimizes damage to surrounding healthy tissues and reduces side effects compared to conventional treatments.^[^ ^1^ ^]^ As a non‐invasive or minimally invasive treatment, PAT reduces the need for surgical interventions, leading to shorter recovery times and decreased patient morbidity. When combined with other treatment modalities, PAT can enhance therapeutic effects. For instance, integrating PDT with immunotherapy has shown synergistic effects in metastatic cancer treatment, improving overall efficacy.^[^ ^2^ ^]^ PAT offers alternative mechanisms to induce cancer cell death, which can be particularly beneficial in cases where tumors exhibit resistance to chemotherapy or radiotherapy. These combined attributes indicate PAT's potential as a versatile and effective tool for cancer therapy.

**Figure 1:**
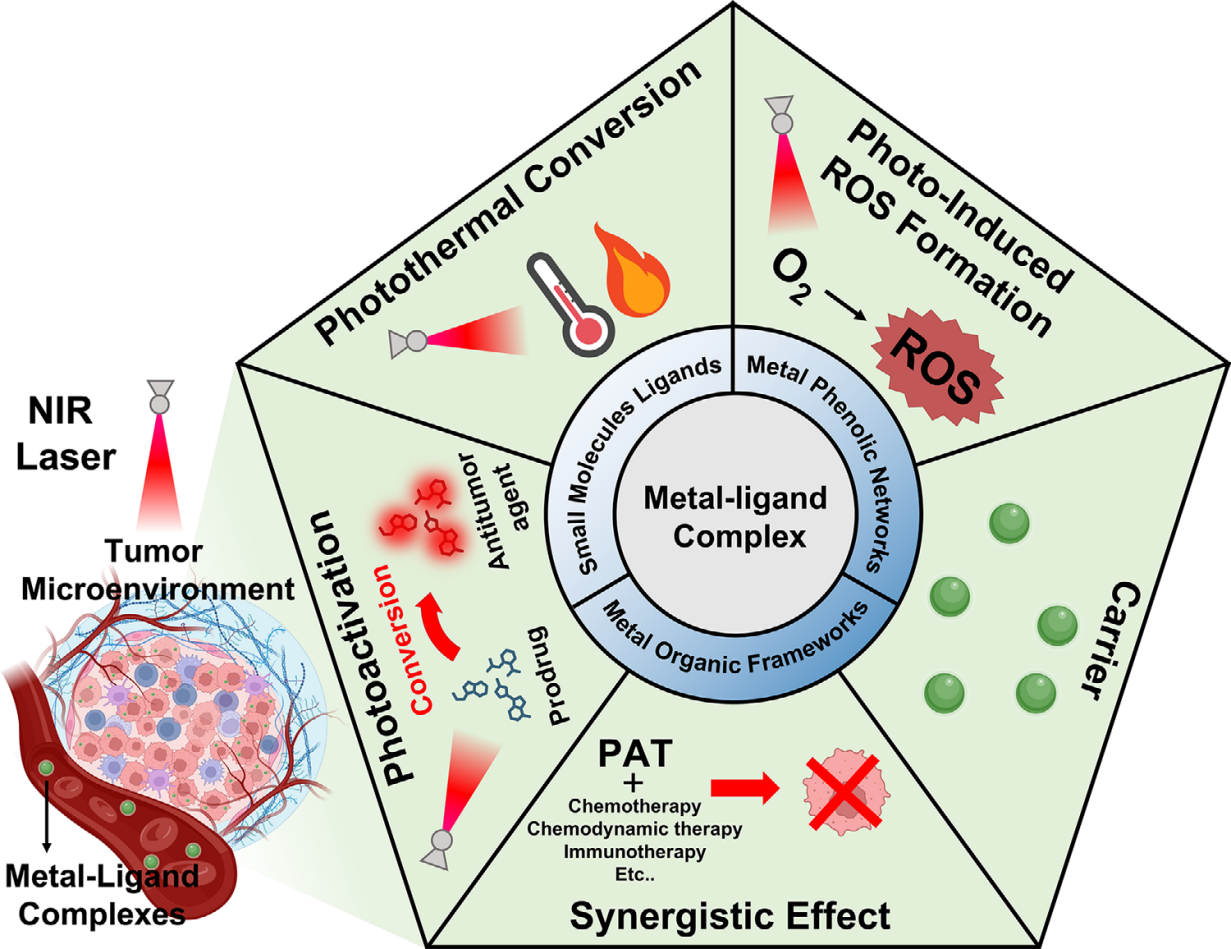
Multifunctional roles of metal‐ligand complexes in photo‐activated cancer therapy. Some images were created with BioRender.com.

Metal‐ligand complexes offer unparalleled versatility through their customizable structures and dynamic physicochemical properties. These complexes are formed by the coordination of metal ions with organic or inorganic ligands, enabling precise control over their optical, electronic, and catalytic characteristics. This adaptability has positioned metal‐ligand complexes as powerful tools in cancer PAT, drug delivery, and imaging.^[^ ^3^, ^4^, ^5^ ^]^ Specifically, metal‐ligand complexes perform multifunctional roles within the tumor microenvironment (TME), including serving as carriers for targeted drug delivery, acting as photosensitizers for ROS generation in PDT, converting NIR light into heat for PTT, and providing photo‐activated conversion for photo‐activated cancer therapy (**Figure** **1**). For instance, their ability to facilitate ligand‐to‐metal charge transfer (LMCT) or metal‐to‐ligand charge transfer (MLCT) mechanisms enhances the generation of reactive oxygen species (ROS) in PDT, crucial for inducing localized cancer cell death. Additionally, the tunable absorption spectrum of metal‐ligand complexes allows for optimization in the near‐infrared (NIR) range, improving penetration depth for PTT and bioimaging. Beyond PAT, these complexes serve as robust platforms for drug delivery, utilizing their high stability and functionalizability to achieve targeted release in the TME. Recent progress in ligand design, including polyphenols and porphyrins, has further expanded their applicability, integrating biocompatibility and specificity into their framework.^[^ ^6^, ^7^ ^]^ These attributes suggest the potential of metal‐ligand complexes in addressing the multifaceted challenges of cancer theranostics.

Numerous review articles in the past five years have discussed the potential of metal‐ligand complexes in cancer diagnosis and therapy.^[^ ^1^, ^2^, ^3^, ^7^, ^8^, ^9^, ^10^, ^11^, ^12^, ^13^, ^14^, ^15^, ^16^, ^17^, ^18^ ^]^ These studies include materials such as metal‐organic frameworks (MOFs), metal‐phenolic networks (MPNs), and various metal‐based nanoparticles. These reviews have provided comprehensive insights into the applications of these complexes in drug delivery and various therapeutic modalities, including PTT, PDT, PACT, and sonodynamic therapy. Additionally, diagnostic techniques such as single photon emission computed tomography, positron emission tomography, and magnetic resonance imaging (MRI) have been extensively discussed in this context.

This review aims to provide a focused analysis of recent advancements in the use of metal‐ligand complexes for PAT, particularly PTT, PDT, and PACT. By systematically categorizing metal ions and ligands, we will elucidate the specific roles of each component and the underlying mechanisms driving the therapeutic efficacy of these systems. Furthermore, we address the inconsistencies in the literature regarding the interchangeable use of PTT, PDT, PACT, and other related modalities such as chemodynamic therapy (CDT). To this end, we propose an alternative explanation based on charge transfer mechanisms and emphasize the need for further mechanistic investigations to clarify these distinctions. In addition to PAT, this review explores broader opportunities for expanding the functionality of ligands used in metal‐ligand complexes. We emphasize the potential of incorporating biologically active ligands, such as nucleic acids and peptides, to achieve dual therapeutic and biological roles. This discussion includes strategies for targeted delivery and the development of long‐term, sustainable delivery systems. We also examine recent progress in ligand discovery and functionalization, emphasizing how these innovations can address challenges in precision medicine and enhance the clinical applicability of metal‐ligand complexes. This review presents recent findings to provide a comprehensive understanding of current research on metal‐ligand complexes in PAT and related fields. It also identifies critical knowledge gaps and proposes directions for future exploration. Ultimately, we aim to bridge the divide between fundamental research and clinical translation, facilitating the development of more effective and versatile therapeutic systems.

## 2 Combination of Diverse Ligands and Metal Ions

The combination of diverse ligands and metal ions allows for the design of highly functional metal‐ligand complexes. These complexes serve as versatile platforms for advanced therapies, demonstrating significant potential in enhancing precision medicine. These customizable properties facilitate a wide range of applications, from PAT to multimodal diagnostics. Such versatility indicates their potential for significant biomedical innovations.

### 2.1 Ligands

**Figure 2:**
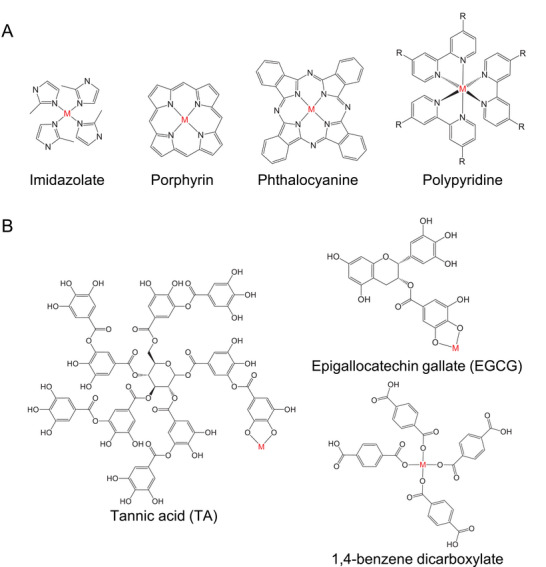
Ligands employed in phototherapy (M = metal), including A) single‐molecule ligands (e.g., porphyrin, phthalocyanine, and polypyridine) and B) macro‐assembly ligands (e.g., TA, EGCG, and terephthalic acid).

Ligands are essential components in the formation of metal‐ligand complexes, providing structural stability and functional versatility. To clarify their distinct roles in applications, ligands can be categorized based on their structural complexity and resulting assembly into two groups: small‐molecule ligands and macro‐assembly ligands (summarized in **Figure** **2**). Small‐molecule ligands typically form discrete metal‐ligand complexes without extended architectures. Representative examples include tetrapyrrolic structures, such as porphyrins, chlorins, and phthalocyanines, known for their excellent photophysical properties. These ligands efficiently absorb NIR light, making them highly suitable as photosensitizers in non‐invasive PDT. Their coordination with metal ions significantly enhances photostability and ROS generation. Additionally, polypyridine ligands have recently attracted considerable attention due to their improved photostability and robust ROS production capabilities upon metal coordination. Macro‐assembly ligands, in contrast, coordinate multiple metal centers to yield extended, 3D structures with distinct physical properties. Notable examples include polyphenols such as tannic acid (TA) and epigallocatechin gallate (EGCG).^[^ ^19^ ^]^ These natural compounds contain multiple phenolic hydroxyl groups, enabling extensive chelation with metal ions to form MPNs.^[^ ^20^, ^21^, ^22^ ^]^ MPNs are highly attractive in biomedical fields due to their pH responsiveness, biocompatibility, low cytotoxicity, and capability to encapsulate therapeutic agents effectively.^[^ ^23^, ^24^ ^]^ Another important class of macro‐assembly ligands includes multidentate organic linkers like terephthalic acid. Due to their linearity and multiple coordination sites, these ligands form MOFs characterized by exceptional porosity and structural tunability, facilitating efficient drug loading and controlled release in therapeutic applications.^[^ ^25^ ^]^

### 2.2 Metal Ions

Metal ions play a critical role in determining the properties and functionality of metal‐ligand complexes. Transition metals and noble metals are frequently employed due to their diverse electronic configurations and reactivity. Transition metals like iron (Fe), manganese (Mn), and copper (Cu) are widely utilized in PAT. For example, Fe(III)‐based complexes are integral to MPNs, enabling NIR light absorption to generate photothermal effects. Beyond this role, Fe(III) can produce ROS through the Fenton reaction and serve as ferroptosis inducers by catalyzing intracellular lipid peroxidation. Mn‐based complexes have demonstrated contributions to photothermal effects, catalytic activity, immune modulation, and T1‐weighted MRI.^[^ ^26^, ^27^ ^]^ Similarly, Cu‐based systems have been investigated for their photothermal capabilities, ROS generation, and imaging applications.^[^ ^28^ ^]^ In addition to these elements, other transition metals such as ruthenium (Ru), iridium (Ir), and rhenium (Re) have demonstrated significant potential as photosensitizers or photoactivatable agents for PDT and PACT applications. Ru(II) complexes exhibit long excited‐state lifetimes, high photostability, efficient ROS generation, and photoinduced ligand dissociation mechanisms, making them attractive for PDT and PACT therapies. Ir(III) complexes are valued for their excellent water solubility, long phosphorescence lifetimes, strong ROS generation, and effective two‐photon excitation. Re(I) complexes also show tumor‐selective ROS production and phototoxicity. Noble metals, such as gold (Au) and platinum (Pt), are commonly used as potent anticancer agents due to their unique photophysical and biochemical properties. Au(III) complexes, in particular, have been developed as PACT agents capable of converting into cytotoxic Au(I) species upon light activation under hypoxic conditions. Similarly, Pt(IV) complexes undergo photoreduction to release active Pt(II) species, enabling spatially and temporally controlled chemotherapy. Collectively, these metals enrich the design space for PAT, offering diverse activation pathways, tunable photophysical features, and adaptable therapeutic strategies suited for next‐generation cancer treatment.

## 3 Optical and Photochemical Properties of Metal‐Ligand Complexes

Metal‐ligand complexes have emerged as highly versatile systems for PDT and other biomedical applications, owing to their unique interaction mechanisms, optical properties, and remarkable capacity to generate ROS. They not only address the limitations of conventional photosensitizers, such as poor water solubility and photostability, but also enable multimodal therapeutic strategies, including combined photothermal and photodynamic therapy. Here, we explore the optical and photochemical properties of metal‐ligand complexes and review the fundamental principles underlying their functionality.

### 3.1 Metal‐Ligand Interaction Mechanisms

The interactions between metals and ligands are primarily governed by coordination bonds, where metal ions, often acting as Lewis acids, coordinate with electron‐rich ligands such as polyphenols, porphyrins, or nitrogen‐based ligands. These interactions enable significant tuning of the physicochemical properties of the complexes. For instance, LMCT and MLCT transitions allow for efficient energy absorption and redistribution, which are critical for ROS generation in PDT.^[^ ^1^ ^]^

In LMCT transitions, electrons move from ligand‐based orbitals to metal‐centered orbitals. This electron transfer is influenced by the ligand's electron‐donating ability and the metal's oxidation state. For instance, complexes with p‐donating ligands, such as oxo groups, can exhibit low‐energy LMCT transitions, leading to strong absorption in the visible region. A classic example is the permanganate ion (MnO_4_^−^), where the intense purple color arises from LMCT transitions involving oxo ligands and manganese. On the contrary, MLCT transitions involve electron transfer from metal‐centered orbitals to ligand‐based orbitals. These transitions are prominent in complexes where the metal has a low oxidation state, and the ligands possess low‐lying p^*^ (antibonding) orbitals. A well‐known example is the Ru(bpy)_3_^2+^ (bpy = bipyridine), which exhibits strong MLCT absorption, making it useful in various photochemical applications.

In PDT, the efficiency of ROS generation is crucial. Upon light irradiation, metal‐ligand complexes can undergo LMCT or MLCT transitions, leading to excited states that interact with molecular oxygen to produce ROS. The nature of the charge transfer influences the type and efficiency of ROS generated. For example, LMCT transitions can facilitate the formation of singlet oxygen (^1^O_2_) or other ROS, depending on the specific metal‐ligand system and the energy states involved. The design of metal‐ligand complexes for PDT applications requires careful selection of both metal centers and ligands to optimize LMCT and MLCT transitions. The ligand's electronic properties, the metal's oxidation state, and the overall geometry of the complex each play a crucial role in shaping its photophysical behavior, collectively impacting the therapeutic efficacy of PDT. Understanding the intricacies of LMCT and MLCT transitions in metal‐ligand complexes enables the development of more effective photosensitizers for PDT, enhancing treatment outcomes through improved ROS generation and targeted cellular interactions.

### 3.2 Optical Properties

Metal‐ligand complexes exhibit exceptional optical properties, making them invaluable in biomedical applications, particularly in PDT and diagnostic imaging. Their tunable electronic structures allow for strong absorption in the visible to NIR spectrum, enabling deeper tissue penetration and minimizing photodamage to healthy cells during treatment.

The ability of metal‐ligand complexes to absorb NIR light is particularly advantageous for non‐invasive therapies. NIR light (700–1700 nm) penetrates biological tissues more effectively than visible light, reducing scattering and absorption by endogenous biomolecules. This property facilitates targeted treatment of deeper‐seated tissues. For instance, lanthanide‐based MOFs have been engineered to exhibit strong NIR luminescence, enhancing their utility in imaging‐guided theranostics.

Many metal‐ligand complexes are designed to emit fluorescence upon excitation, serving as effective agents for imaging applications. Lanthanide complexes, for example, are known for their sharp emission bands and long luminescence lifetimes, which are beneficial for time‐resolved fluorescence imaging. These properties enable high‐contrast imaging with minimal background interference, crucial for accurate diagnostics.

Beyond fluorescence, metal‐ligand complexes can be utilized in photoacoustic imaging – a hybrid technique combining optical and ultrasound imaging. Upon absorption of pulsed laser light, these complexes generate ultrasonic waves due to rapid thermoelastic expansion. This mechanism allows for high‐resolution imaging of biological tissues. For example, certain MOFs have been explored for their potential in photoacoustic imaging, providing detailed insights into tissue structure and function.

The optical properties of metal‐ligand complexes can be finely tuned by modifying the metal centers and ligand environments. Incorporating heavy metals like iridium (Ir) or ruthenium (Ru) can enhance spin‐orbit coupling, leading to increased intersystem crossing and improved generation of ROS for PDT. Similarly, altering ligand structures can shift absorption and emission wavelengths, optimizing the complexes for specific therapeutic or diagnostic needs. Therefore, the versatile optical properties of metal‐ligand complexes, including NIR absorption, fluorescence emission, and photoacoustic signal generation, indicate their significant potential as dual diagnostic and therapeutic agents.

### 3.3 Mechanisms of Photo‐Activated Therapy

**Figure 3:**
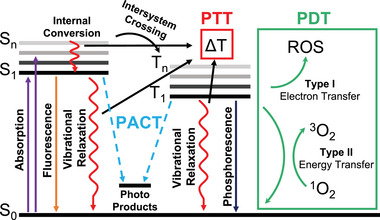
A simplified Jablonski diagram illustrating the mechanisms of PTT, PDT, and PACT. When a molecule is exposed to photons with energies greater than its energy gap, an electron in the ground singlet state (S_0_) can be excited to an excited singlet state (S_1_ or S_n_). The electron then undergoes vibrational relaxation to S_1_ and may return to the ground state (S_0_) either through radiative decay, emitting energy as fluorescence, or through non‐radiative decay, releasing energy as heat. Alternatively, the electron in the S_1_ state may transition to a lower‐energy triplet state (T_1_) via ISC. The unstable T_1_ state can decay back to the ground state (S_0_) by releasing heat or through phosphorescent emission. The triplet excited state can also transfer energy to nearby triplet oxygen, generating cytotoxic singlet oxygen that kills cancer cells through the type‐II process. Additionally, the triplet molecule can donate electrons to biological substrates, producing harmful type‐I radicals (ROS). In PACT, cytotoxic species are produced via photochemical pathways, including ligand dissociation and redox reactions that occur in the excited states of metal‐ligand complexes.

Cancer cells can be effectively killed when exposed to temperatures exceeding 42°C.^[^ ^29^, ^30^ ^]^ PTT utilizes the unique ability of photothermal agents, such as noble metal nanoparticles, metal‐ligand complexes, and photosensitizers, to absorb light and release energy through non‐radiative relaxations, resulting in localized temperature increases for therapeutic purposes (**Figure** **3**).^[^ ^31^ ^]^ This method offers several advantages, including its non‐invasive nature, applicability to drug‐resistant tumors, ability to induce immediate and localized effects, and potential for synergistic integration with other treatment modalities to maximize therapeutic efficacy.^[^ ^32^ ^]^ NIR light is predominantly employed due to its high tissue penetration capability, enabling energy transfer to internal photothermal agents and achieving localized temperature elevation to effectively ablate cancer cells.^[^ ^10^, ^33^ ^]^

PDT utilizes metal‐ligand complexes as photosensitizers to generate ROS through two primary mechanisms: Type I and Type II. Both mechanisms involve the transition of the photosensitizer to an excited triplet state upon light absorption. This process is facilitated by the heavy atom effect of the metal center, which enhances intersystem crossing (ISC) from the singlet to the triplet state, as shown in Figure 3.

1. Type‐I Mechanism: In this pathway, the excited triplet state photosensitizer undergoes electron or hydrogen transfer interactions with surrounding substrates, leading to the formation of radical species. These radicals subsequently react with molecular oxygen and water to produce superoxide anions (O_2_^•−^) and hydroxyl radicals (⋅OH). These ROS are highly reactive and can induce significant oxidative damage to cellular components, including lipids, proteins, and nucleic acids, ultimately leading to cell death. This mechanism is particularly advantageous in hypoxic tumor environments where oxygen levels are low, as it does not rely solely on molecular oxygen for ROS generation.
2. Type‐II Mechanism: The excited triplet state photosensitizer transfers its energy directly to ground‐state molecular oxygen, converting it into singlet oxygen, a highly cytotoxic species known for its role in initiating apoptosis or necrosis. This mechanism is oxygen‐depedent and is most effective in well‐oxygenated tissues.

Both pathways rely on the triplet state of the photosensitizer for effective ROS generation, highlighting the critical role of photophysical properties in designing efficient PDT agents. Metal‐ligand complexes are particularly effective in PDT due to their tunable photophysical properties. Selecting appropriate metals and ligands significantly impacts photophysical properties. Specifically, these choices affect the efficiency of intersystem crossing, the lifetime of the triplet excited state, and the quantum yield of ROS generation. For instance, Ir(III) complexes have been developed as Type I dominant photosensitizers, capable of generating hydroxyl radicals upon irradiation, making them suitable for hypoxic tumor treatment. Understanding the balance between Type I and Type II mechanisms is crucial for optimizing PDT efficacy, especially considering the varying oxygenation levels in different TMEs. The design of metal‐ligand complexes that can operate effectively under both normoxic and hypoxic conditions remains a significant focus in the development of new photosensitizers for cancer therapy.

Photo‐activated chemotherapy (PACT) represents a targeted therapeutic approach in which biologically inactive prodrugs, typically metal‐ligand complexes, are selectively activated at tumor sites upon exposure to specific wavelengths of light. Upon irradiation, these metal‐ligand prodrugs transition from their electronic ground state into excited singlet or triplet states, resulting in structural rearrangements or changes in electronic configuration. Generally, activation occurs through two primary pathways: ligand photorelease and ligand photoactivation, each mediated by distinctive photochemical mechanisms.^[^ ^34^ ^]^

In the photorelease pathway, therapeutic activation typically occurs through either photoinduced redox reactions or photosubstitution reactions. The photoinduced redox reactions involve electron‐transfer processes between metal centers and coordinated ligands upon photoactivation, resulting in photoreduction or photooxidation. Consequently, cytotoxic ligands or reactive metal species are selectively released from the parent complex. Alternatively, in the photosubstitution pathway, light irradiation triggers ligand substitution without significant electron transfer, where a coordinated ligand is replaced by a solvent molecule. Due to the inherently weaker metal‐solvent bonds, these newly formed intermediates are particularly reactive and readily interact with biomolecules within tumor cells, inducing targeted cytotoxic effects.^[^ ^34^ ^]^ Both photorelease mechanisms effectively yield active species capable of directly damaging critical biological targets, including DNA, RNA, or proteins, or promoting the localized generation of bioactive signaling molecules such as nitric oxide or carbon monoxide, thereby amplifying therapeutic efficacy.^[^ ^35^ ^]^ In contrast, the ligand photoactivation mechanism involves direct photochemical transformations of the ligand itself, such as photocleavage or photoswitching, upon absorption of light. These photo‐activated ligands transition from inert or minimally cytotoxic forms to highly cytotoxic structures, thereby directly exerting therapeutic effects without the necessity of ligand dissociation from the metal center. Recent developments have demonstrated the effectiveness of ligand photoactivation in metal‐ligand complexes, highlighting their potential to significantly enhance selectivity and therapeutic outcomes in cancer treatment.^[^ ^36^ ^]^

Although PTT, PDT, and PACT primarily involve photo‐activation, their underlying mechanisms differ distinctly and can synergize effectively. PDT typically generates ROS such as singlet oxygen, superoxide anions, and hydroxyl radicals, depending on the photosensitizer type and excitation pathway (Type I or Type II mechanisms). PTT converts absorbed NIR light into localized heat through non‐radiative relaxation, causing hyperthermia‐induced tumor destruction.^[^ ^37^ ^]^ PACT leverages light to activate inert metal‐based prodrugs, enabling controlled release of cytotoxic species at the tumor site. The thermal effect of PTT can sensitize cancer cells, enhancing susceptibility to ROS‐induced apoptosis or necrosis triggered by PDT, leading to synergistically improved therapeutic outcomes.^[^ ^38^ ^]^ Furthermore, the localized temperature elevation from PTT increases blood flow and improves hemoglobin oxygen saturation in tumors, mitigating hypoxia, thus potentiating the efficacy of oxygen‐dependent Type II PDT processes.^[^ ^39^, ^40^ ^]^ Also, the PTT‐induced heat can promote photoactivatable drug release, thereby enhancing the therapeutic efficacy of PACT.^[^ ^41^, ^42^ ^]^ Chemotherapy and CDT further complement these phototherapies. Oxygen consumption during the ROS generation process of PDT can improve the chemotherapy efficiency of the hypoxia‐activated prodrug (e.g., tirapazamine, banoxantrone, and evofosfamide).^[^ ^43^, ^44^, ^45^ ^]^ CDT exploits Fenton or Fenton‐like reactions, often mediated by iron ions, to convert endogenous hydrogen peroxide (H_2_O_2_) into highly reactive hydroxyl radicals, independent of external light stimuli.^[^ ^46^, ^47^ ^]^ PDT‐generated ROS can elevate cellular oxidative stress, increasing intracellular hydrogen peroxide availability, which enhances CDT efficiency by providing additional substrates for Fenton reactions. Ferroptosis, a distinct iron‐dependent form of regulated cell death driven primarily by lipid peroxidation, intersects closely with both PDT and CDT.^[^ ^48^ ^]^ The ROS generated by PDT and CDT initiate and propagate lipid peroxidation in cancer cells, especially when iron redox cycling occurs, where iron ions alternate between ferrous (Fe^2+^) and ferric (Fe^3+^) states, perpetuating oxidative stress and lipid radical formation.^[^ ^49^, ^50^ ^]^ These interconnected mechanisms provide multiple synergistic pathways for tumor eradication. For instance, PDT‐generated ROS amplifies CDT and ferroptosis by promoting iron redox cycling and lipid oxidation, while PTT‐induced hyperthermia simultaneously enhances cellular susceptibility to oxidative stress and disrupts cellular antioxidant defenses, ultimately amplifying overall treatment efficacy.

### 3.4 Cell Death Pathways in Photo‐Activated Therapy

PAT modalities – PTT, PDT, and PACT – are known to activate multiple RCD pathways within cancer cells, notably apoptosis, necroptosis, pyroptosis, and ferroptosis, among others.^[^ ^51^, ^52^ ^]^ Typically, PDT induces apoptosis primarily through ROS‐mediated damage to critical cellular components such as DNA and mitochondria, triggering caspase‐dependent apoptotic cascades.^[^ ^53^ ^]^ Similarly, localized hyperthermia induced by PTT has been shown to provoke apoptotic cell death via protein denaturation, unfolded protein responses, and activation of stress signaling pathways. In the context of PACT, photoactivation of chemotherapeutic agents typically results in direct DNA damage, thereby initiating apoptotic signaling.^[^ ^54^, ^55^ ^]^ However, when apoptosis induction is insufficient due to excessive oxidative damage, impaired signaling, or activation of anti‐apoptotic pathways, cells may undergo caspase‐independent alternative forms of cell death such as necroptosis.^[^ ^56^, ^57^ ^]^ Necroptosis is characterized by cellular swelling, organelle disruption, membrane rupture, and subsequent cell lysis, which releases intracellular contents and danger signals to neighboring immune cells, potentially enhancing immunogenicity and antitumor immune responses.^[^ ^58^ ^]^ In addition, PAT‐induced cellular injury may activate inflammatory caspases or promote the formation of gasdermin‐mediated pores, leading to pyroptosis, a highly inflammatory type of RCD.^[^ ^59^ ^]^ For example, ROS generated by PDT directly triggers cellular damage, which activates caspase‐1 or caspase‐8/3, subsequently causing pyroptotic cell lysis and enhancing local inflammation.^[^ ^60^ ^]^ Similarly, heat stress from PTT stimulates the release of damage‐associated molecular patterns (DAMPs), promoting inflammasome formation and caspase‐1‐dependent cleavage and activation of gasdermin D. Moreover, drug‐induced oxidative stress resulting from PACT can activate caspase‐3 pathways, facilitating a cell‐death transition from apoptosis to pyroptosis.^[^ ^61^, ^62^ ^]^

Ferroptosis, a distinct iron‐dependent, lipid‐peroxidation‐driven form of RCD, can also be effectively triggered by PAT. For instance, ROS generated during PDT can directly initiate lipid peroxidation, while elevated local temperatures caused by PTT can enhance intracellular enzymatic reactions, accelerating iron‐catalyzed Fenton reactions and further amplifying lipid peroxide formation.^[^ ^63^, ^64^ ^]^ Additionally, photo‐activated drugs in PACT can induce ferroptosis by disrupting cellular redox balance; for example, the Ru(II)‐sorafenib (SRF) prodrug releases SRF upon illumination, leading to glutathione (GSH) depletion and inhibition of glutathione peroxidase 4 (GPX4), ultimately resulting in ferroptotic cell death.^[^ ^65^ ^]^

Despite the potential of PAT to induce diverse and potent cell‐death mechanisms, these modalities still possess several significant limitations. A critical challenge arises from the oxygen dependency of ROS generation in PDT, which markedly reduces treatment efficacy in hypoxic TMEs commonly encountered in solid tumors.^[^ ^66^ ^]^ Furthermore, tumor cells can develop resistance to apoptosis through multiple mechanisms such as increased expression of anti‐apoptotic genes, enhanced antioxidant defenses, and drug efflux mechanisms, thereby limiting the efficacy of PAT strategies that rely solely on a single RCD pathway.^[^ ^67^ ^]^ To address these challenges, recent works have increasingly focused on dual‐modality PAT approaches that simultaneously exploit multiple distinct RCD pathways. These combination strategies, such as simultaneous activation of apoptosis and ferroptosis or pyroptosis, offer significant advantages in overcoming apoptotic resistance and enhancing therapeutic efficacy. Among these approaches, metal‐ligand complexes have attracted particular attention, as the controlled release of metal ions upon photoactivation effectively triggers ferroptosis in combination with other PAT‐induced RCD pathways, substantially improving cancer treatment outcomes.^[^ ^68^ ^]^

## 4 Metal‐Ligand Complexes for Photothermal Therapy

Research on metal‐ligand complexes as photothermal agents has mainly targeted two goals: optimizing NIR absorption for effective treatment of deep tumors, and improving photothermal conversion efficiency to generate more heat with equal or less energy. To reach these goals, various ligands have been designed to promote strong MLCT and LMCT transitions, refine molecular geometry for better energy transfer, and add functional groups that boost stability and biocompatibility. Recent advances show that hybrid metal‐ligand complexes surpass traditional nanoparticles in photothermal efficiency and enable combined cancer therapies. This section reviews the latest developments in metal‐ligand complexes for PTT, highlighting how metal‐ligand coordination enhances therapeutic performance. **Table** **1** summarizes key studies involving different ligand and metal ion combinations.

**Table 1:** Overview of metal‐ligand complexes in PTT and complementary cancer therapies: detailing therapy types, metal ions, ligands, roles in therapy, platform sizes, tumor models, target diseases, and light sources for activation.

Therapy type	Metal ion	Ligand	Role of metal‐ligand complex	Size	Tumor model	Disease	Light source	References
PTT	Fe(III)	TA	Photothermal agent	196 nm	4T1 tumor‐bearing mice	Cancer	808 nm	[69]
PTT, immunoth‐erapy	Fe(III)	TA	Photothermal agent, antibody carrier	100–200 nm	4T1 tumor‐bearing mice	Cancer	808 nm	[70]
PTT, immunoth‐erapy	Fe(III)	Gallic acid	Photothermal agent, antibody carrier	193.5 nm	4T1 tumor‐bearing mice	Cancer	808 nm	[71]
PTT, immunoth‐erapy	Fe(III)	Phenolic‐ semicond‐uctor polymer	Fe (III) ion carrier (ferroptosis)	80 nm	B16–F10 tumor‐bearing mice model	Cancer	808 nm	[72]
PTT	Mn(IV)	Hexahydr‐azide clathroch‐elate ligand	Photothermal agent	4.19 nm	4T1 tumor‐bearing mice	Cancer	730 nm	[73]
PTT, chemothe‐rapy	Cu(II)	Benzened‐icarboxylic acids	Cu (II) ion carrier (ferroptosis)	200 nm	4T1 tumor‐bearing mice	Cancer	1064 nm	[74]
PTT	Ni(II)	Thiophenes	Photothermal agent	98.5–109.1 nm	4T1 tumor‐bearing mice	Cancer	1064 nm	[75]
PTT, chemothe‐rapy	Fe(III)	Luteolin	Photothermal agent, pH‐ sensitive carrier	161 nm	‐	Cancer	660 nm	[76]
PTT, photo‐Fenton CDT	Fe(III)	TA	Photothermal agent	54 nm	U14 tumor‐bearing mice, HeLa cells	Cancer	980 nm	[77]
PTT, CDT	Fe(III)	TA	Carrier	120 nm	4T1 tumor‐bearing mice, 4T1 cells	Cancer	1064 nm	[78]

### 4.1 Metal‐Phenolic Networks for Photothermal Therapy

MPNs have been extensively investigated as drug delivery carriers since their first introduction by the Frank Caruso group in 2013. These materials are highly attractive due to their simple and rapid synthesis process and their pH‐sensitive stability, which enables effective delivery of therapeutic agents.^[^ ^3^, ^79^ ^]^ In 2018, the Feng group demonstrated that MPNs could exhibit absorption shifts from the UV‐visible range to the far‐red to NIR region due to LMCT transitions.^[^ ^69^ ^]^ For instance, when TA complexes with Fe^3+^, V^3+^, or Ru^3+^, the absorption range of TA shifts from the UV region to 500–900 nm, allowing the absorption of 808 nm laser light and subsequent conversion of light into heat energy. This system achieved a photothermal conversion efficiency of up to 40% and elevated local temperatures to as high as 70°C, depending on concentration and irradiation duration. Beyond PTT, MPN particles were also shown to be effective for photothermal imaging. Huang et al. (2021) developed poly(lactic‐co‐glycolic acid) (PLGA) nanoparticles coated with MPNs formed using TA and Fe^3+^ ions.^[^ ^70^ ^]^ The resulting PLGA‐MPN nanoparticles demonstrated strong PTT properties and were used to simultaneously achieve PTT and immunotherapy. These nanoparticles absorbed a broad range of light from 300 to 800 nm, effectively converting 808 nm laser energy into heat, raising cancer cell temperatures to 60°C, and significantly suppressing tumor growth. Although the photothermal conversion efficiency was not precisely calculated, the system effectively eradicated cancer cells. Moreover, combining the nanoparticles with an anti‐programmed death‐ligand 1 (PD‐L1) antibody completely inhibited tumor growth and metastasis.

Given the versatility of MPNs as drug delivery carriers, studies have explored their application in synergistic therapies by incorporating payloads compatible with immunotherapy.^[^ ^71^ ^]^ Gallic acid, for example, forms MPNs through coordination with Fe^3+^ ions, while also interacting with proteins via hydrogen bonding. In mildly alkaline conditions, mixing Fe^3+^ ions, GA, and proteins such as ovalbumin (OVA), human serum albumin (HSA), or bovine serum albumin (BSA) yields respective OVA‐Fe‐GA, HSA‐Fe‐GA, and BSA‐Fe‐GA nanoparticles. Among these, BSA‐Fe‐GA nanoparticles combine the immune‐activating properties of BSA with the PTT capabilities of Fe‐GA complexes. These nanoparticles exhibit broad absorption from 400 to 800 nm and achieve a photothermal conversion efficiency of up to 20% under 808 nm laser irradiation. In vivo studies showed that BSA‐Fe‐GA nanoparticles effectively suppressed tumor growth in a 4T1 tumor model by elevating tumor temperatures and facilitating immune activation.

**Figure 4:**
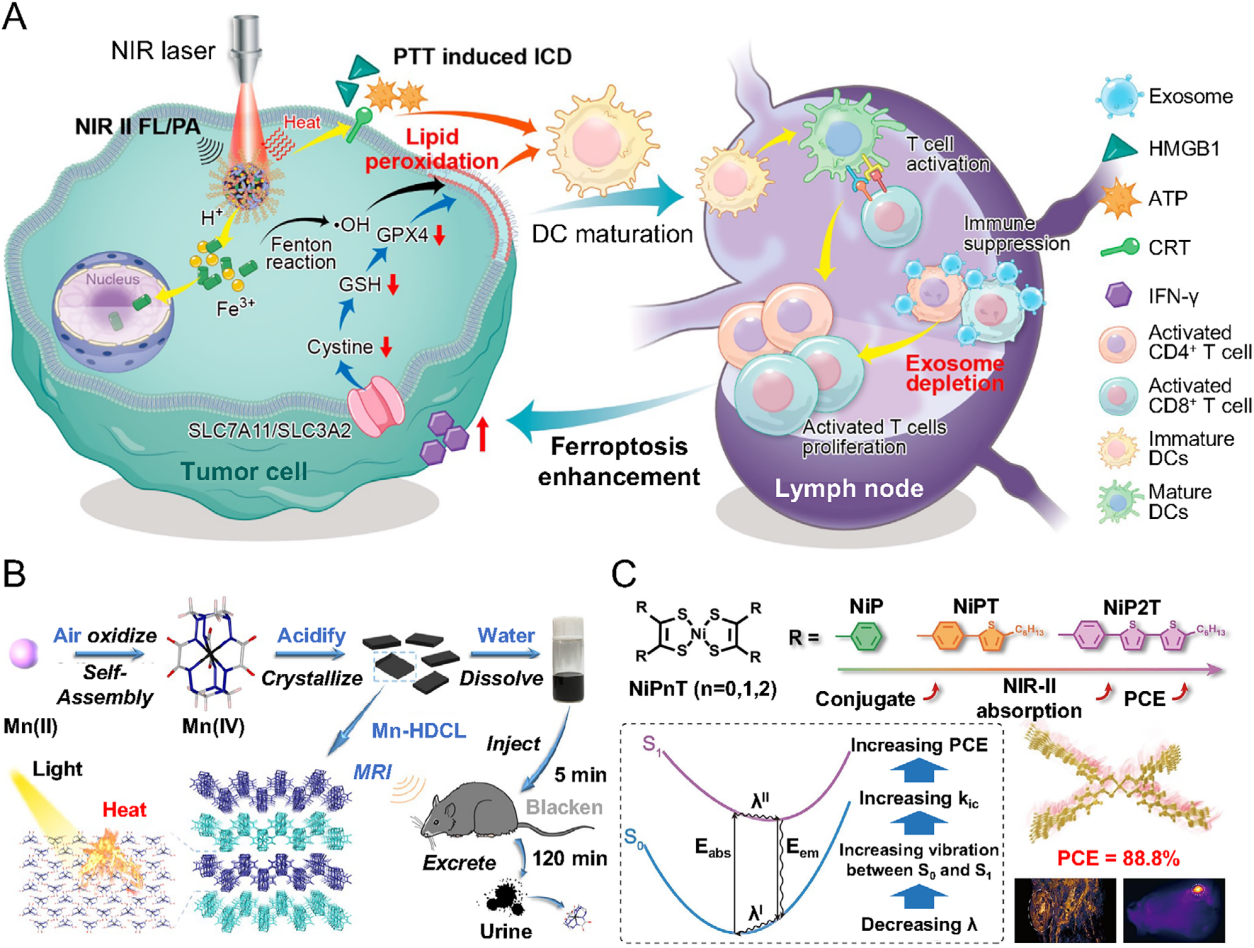
Representative studies on metal‐ligand complexes for PTT. A) Illustration of the PFG MPNs’ PTT combined with ferroptosis. Reproduced with permission.^[^ ^72^ ^]^ Copyright 2022, American Chemical Society. B) Schematic of Mn‐HDCL, incorporating a hexahydrazide clathrochelate ligand and Mn^4+^ ions. Reproduced with permission.^[^ ^73^ ^]^ Copyright 2022, American Chemical Society. C) Representation of ligand field modulation in organonickel(II) complexes (NiPnT, n = 0, 1, 2). Reproduced with permission.^[^ ^75^ ^]^ Copyright 2025, Elsevier.

MPNs can also incorporate metal ions capable of inducing ferroptosis, providing the opportunity to combine PTT with metal ion‐mediated ferroptosis. Fe^3+^ ions, in particular, generate ROS through the Fenton reaction, inhibiting tumor growth and enabling CDT.^[^ ^80^ ^]^ Xie et al. (2022) developed phototheranostic MPNs (PFG‐MPNs) using Fe^3+^ ions, a phenolic polymer, polyethylene glycol (PEG) polymer, and GW4869 (**Figure** **4**A).^[^ ^72^ ^]^ These PFG‐MPNs integrate multiple functions, including PTT, ferroptosis induction through metal‐phenolic coordination, and immunogenicity regulation by GW4869, an exosome inhibitor. PFG‐MPNs enhance immunogenic cell death (ICD) through PTT, promote dendritic cell (DC) maturation, and activate T cells while reinforcing ferroptosis. With a broad absorption range of 600–1000 nm enabled by the semiconductor polymer, PFG‐MPNs achieved a remarkable photothermal efficiency of 75.6% under 808 nm laser irradiation. This synergistic approach showed a profound antitumor effect by combining PTT, ferroptosis, and immunotherapy.

Recently, Leng et al. (2025) developed a multifunctional theranostic platform using Au nanorods coated with Fe^3+^‐TA‐based MPNs, termed Au nanorods@TA‐Fe.^[^ ^77^ ^]^ This platform replaced conventional surface ligands, such as cetyltrimethylammonium bromide, improving biocompatibility. Further surface functionalization with mercaptofolic acid allowed selective tumor targeting. In the TME, Fe‐TA complexes released iron ions, initiating a Fenton reaction to produce cytotoxic hydroxyl radicals, effectively achieving CDT. Additionally, the platform exhibited strong photothermal conversion capability and photoacoustic imaging performance in the NIR‐II region, enhancing tumor targeting and therapy guidance. Qu et al. (2025) introduced core‐shell nanoparticles (NYF:Er, Ce, Tm@CaF_2_) surface‐modified with Fe‐TA MPNs (NYF:Er, Ce, Tm@CaF_2_@Fe‐TA).^[^ ^78^ ^]^ These nanoparticles displayed dual upconversion/downconversion properties, converting NIR to UV emissions and NIR‐II downconversion emissions. Under UV illumination, Fe^3+^ was reduced to Fe^2+^, continuously supplying Fe^2+^ for a photo‐Fenton‐like CDT effect, while simultaneously demonstrating excellent photothermal effects with a 980 nm laser. This multifunctional platform also enabled MRI‐guided therapy, indicating its promising potential for simultaneous tumor imaging and treatment.

### 4.2 Metal‐Organic Frameworks for Photothermal Therapy

Recent research has been actively exploring the use of MOFs for PTT due to their tunable porosity and excellent photothermal conversion properties. MOF‐based PTT systems can be designed to incorporate photoactive metal centers and organic linkers, enabling efficient light absorption and heat generation under NIR irradiation. Additionally, MOFs offer the advantage of drug loading and controlled release, making them promising candidates for synergistic cancer therapy by combining PTT with chemotherapy or immunotherapy. Xu et al. (2022) synthesized a Mn^4+^ complex with high absorption across the UV–vis–NIR spectrum, pioneering its application in MRI and PTT (Figure 4B).^[^ ^73^ ^]^ By utilizing a hexahydrazide clathrochelate ligand (L/HDCL), they developed a sheet‐like 2D Mn‐HDCL complex. The Mn‐HDCL complex exhibited enhanced NIR absorption and a large extinction coefficient due to MLCT. This complex effectively absorbed light across a broad spectrum from 200 to 1000 nm and demonstrated superior absorption at 800 nm compared to single‐walled carbon nanotubes. Upon exposure to an 808 nm laser, the Mn‐HDCL complex achieved a photothermal conversion efficiency of ≈71%. It selectively elevated tumor temperatures to 60°C in vivo and significantly suppressed the growth of 4T1 tumors.

Geng et al. (2022) reported CuS decorated Cu‐based MOF (CuS@Cu‐MOF) with excellent hydrophilicity, high absorption performance in an NIR region, fenton‐like catalytic performance, and high drug loading efficiency.^[^ ^74^ ^]^ Cu^2+^ ions and benzenedicarboxylic acids were used to prepare the Cu‐MOF nanocomposites. A partial in situ vulcanization method was used for the in situ growth of CuS nanoparticles (≈4 nm) on Cu‐MOF. Doxorubicin (DOX) loaded into the CuS@Cu‐MOF, coated with amphiphilic 1, 2‐distearoyl‐sn‐glycero‐3‐phosphoethanolamine (DSPE)‐PEG molecules to improve hydrophilicity. CuS@Cu‐MOF nanocomposites showed enhanced NIR light absorption and excellent photothermal conversion efficiency (39.6%) due to the plasmonic properties of CuS. Also, the Cu ions, which were released from CuS@Cu‐MOF, enable a Fenton‐like reaction (H_2_O_2_ → •OH), with the reaction rate further boosted by 1.53 times through photothermal effects. The porous structure of CuS@Cu‐MOF allowed efficient drug loading (DOX, 25.5%), with pH‐responsive release in acidic tumor environments, thereby improving chemotherapy outcomes.

### 4.3 Other Metal‐Ligand Complexes for Photothermal Therapy

Chen et al. (2025) investigated Ni^2+^‐ligand complexes to optimize their ligand field, thereby enhancing absorption spectra and photothermal conversion efficiency.^[^ ^75^ ^]^ Typically, ligand‐induced splitting of a metal's d orbitals creates high separation energies that limit light absorption. Chen et al. engineered the ligand structure in Ni^2+^‐ligand complexes to modulate the ligand field, expanding conjugation and promoting exciton transfer. This strategy enhanced NIR II (900–1600 nm) photoreactivity. In the NiPnT complexes (n = 0, 1, or 2), increasing the number of thiophene (T) groups extended resonance structures, reduced reorganization energy, and increased non‐radiative transition rates by lowering ligand field separation energies (Figure 4C). As a result, NiPnT nanoparticles achieved a remarkable photothermal conversion efficiency of 88.8% under a 1064 nm laser and demonstrated efficient NIR II absorption (800–1200 nm). In vivo experiments showed that NiPnT nanoparticles selectively raised tumor temperatures to 55°C, effectively inhibiting tumor growth in 4T1 models.

Natural compounds have also been explored for PTT applications, with studies investigating their potential as cancer therapeutics. Ruan et al. (2024) developed a luteolin‐Fe^3+^ complex for chemo‐photothermal therapy.^[^ ^76^ ^]^ By combining luteolin, Fe^3+^ ions, and mPEG‐b‐PLGA using flash nanoprecipitation, they synthesized Luteolin/Fe^3+^/mPEG‐b‐PLGA (LPP) nanoparticles. Unlike luteolin alone, the LPP nanoparticles exhibited broad absorption from 500 to 800 nm due to LMCT between luteolin and Fe^3+^. When exposed to a 660 nm laser, LPP nanoparticles raised temperatures to a maximum of 90°C. Although the photothermal conversion efficiency was not reported, the nanoparticles retained PTT efficacy under acidic conditions (pH 5.5 and pH 6.6) and released luteolin at lower pH, indicating potential for tumor‐specific therapy.

These works exhibit the versatility of metal‐ligand complexes in expanding the capabilities of PTT. From engineered ligand fields to natural compound‐based systems, such developments demonstrate significant potential for improving therapeutic precision and efficiency.

## 5 Metal‐Ligand Complexes for Photodynamic Therapy

PDT is a medical technique recognized for its high temporal and spatial selectivity, particularly in cancer treatment due to its specificity, non‐invasive nature, and low side effects.^[^ ^81^, ^82^ ^]^ However, PDT efficacy is hindered by poor photostability of photosensitizers, limited tissue penetration of light, and the suppressive nature of the TME.^[^ ^83^ ^]^ To address these challenges, metal‐ligand complexes have been explored as both photosensitizers and carriers for PDT. These complexes can boost ROS production through catalytic mechanisms while also enhancing light absorption and structural stability. This section highlights recent advances in using metal‐ligand complexes in PDT, emphasizing their roles and potential to address current therapeutic limitations.

### 5.1 Metal‐Ligand Complexes as a Photosensitizer for PDT

The integration of metal ions into photosensitizers enhances their photostability, decreases photobleaching, improves biocompatibility, and allows for structural modification. Metal‐based photosensitizers often possess large two‐photon absorption cross sections, benefiting NIR applications.^[^ ^84^ ^]^ For example, Ru complexes offer long excited‐state lifetimes, two‐photon excitation capabilities, and high selectivity, while Ir complexes provide excellent water solubility, long phosphorescence lifetimes, and efficient ROS generation even in hypoxic conditions. Conventional polypyrrole‐based systems and MOFs with porphyrin linkers utilize MLCT or LMCT transitions to produce ROS. This section reviews recent advances, highlighting sophisticated molecular designs that exploit the distinct benefits of metal complexes. **Table** **2** provides an overview of recent relevant studies.

**Table 2:** Summary of metal‐ligand complexes as photosensitizers in PDT and complementary cancer therapies: outlining therapy types, metal ions, ligands, roles in therapy, platform sizes, tumor models, target diseases, and light sources for activation.

Therapy type	Metal ion	Ligand	Role of metal‐ligand complex	Size	Tumor model	Disease	Light source	References
PDT	Ru(II)	Indoline‐pyridoben‐zopyrans	Photosensitizer	‐	4T1 tumor‐bearing mice, MCF‐7 cells	Cancer	660 nm	[85]
PDT	Zn(II), Zr(IV)	Protoporphyrin IX for Zn(II), amino‐terphthalic acid ligand, and phosphate of DNA for Zr(IV)	Photosensitizer and carrier	200 nm	MDA‐MB‐231 tumor‐bearing mice, MDA‐MB‐231 and OVCAR‐3 cells	Cancer	532 nm	[86]
PDT	Ir(III)	Pyridine‐based cyclometalated ligands	Photosensitizer	∼100 nm	Balb/C mice subcutan‐eously inoculated with 4T1 cancer cells, 4T1 cancer cells	Cancer	White light	[87]
PDT	Ru(II)	Polypyridyl	Photosensitizer	‐	HT‐29, CT‐26, HeLa, and A2780 cells	Cancer	740 nm	[88]
PDT	Re(I), Ir(III), Pt(II), and Rh(III)	Rhodamine‐tethered bipyridine	Photosensitizer	‐	MCF‐7 tumor‐bearing mice, 4T1, A549, and MCF‐7 cells	Cancer	Visible light	[89]
PDT	Ir(III)	Acetylacetonate	Photosensitizer	‐	HeLa cells	Cancer	Visible light	[90]
PDT	Fe(III), Zr(IV)	Porphyrin	Fe(III) for photosensitizer, Zr(IV) for carrier frame	∼81.6 nm	U87MG tumor‐bearing mice, U87MG cells	Cancer	980 nm	[91]
PDT	Zn(II), Pt(II)	Tert‐butylsulf‐onyl‐substituted phthalocyanines	Photosensitizer	27.77 and 23.88 nm for Zn(II) and Pt(II) complexes encapsula‐ted in micelles, respective‐ly	Human‐induced pluripotent stem cells‐derived colonic cancer organoids, CT26‐bearing mice, A549, LLC, and CT26 cells	Colorectal cancer	635 nm	[92]
Two‐photon PDT, CDT	Ir(III), Fe(III)	Polyphenol‐modified bipyridine ligands	Ir(III) for photosensitizer, Fe(III) for ferroptosis	79 nm	A375 tumor‐bearing mice	Melanoma	730 nm two‐photon irradiation	[93]
Two‐photon PDT	Ir(III)	Thiol functional‐ized bipyridine ligand	Photosensitizer	84 nm	A549 tumor‐bearing mice, A549 cells	Cancer	720 nm two‐photon irradiation	[94]
PDT, CDT	Cu(II), Fe(III)	Gallic acid, 2‐aminoterephthalic acid	Photosensitizer	225 nm	U14 tumor‐bearing mice, HeLa cells	Cancer	650 nm	[95]

#### 5.1.1 Tailoring Ligands in Metal‐Ligand Complexes for Enhanced Photodynamic Therapy

**Figure 5:**
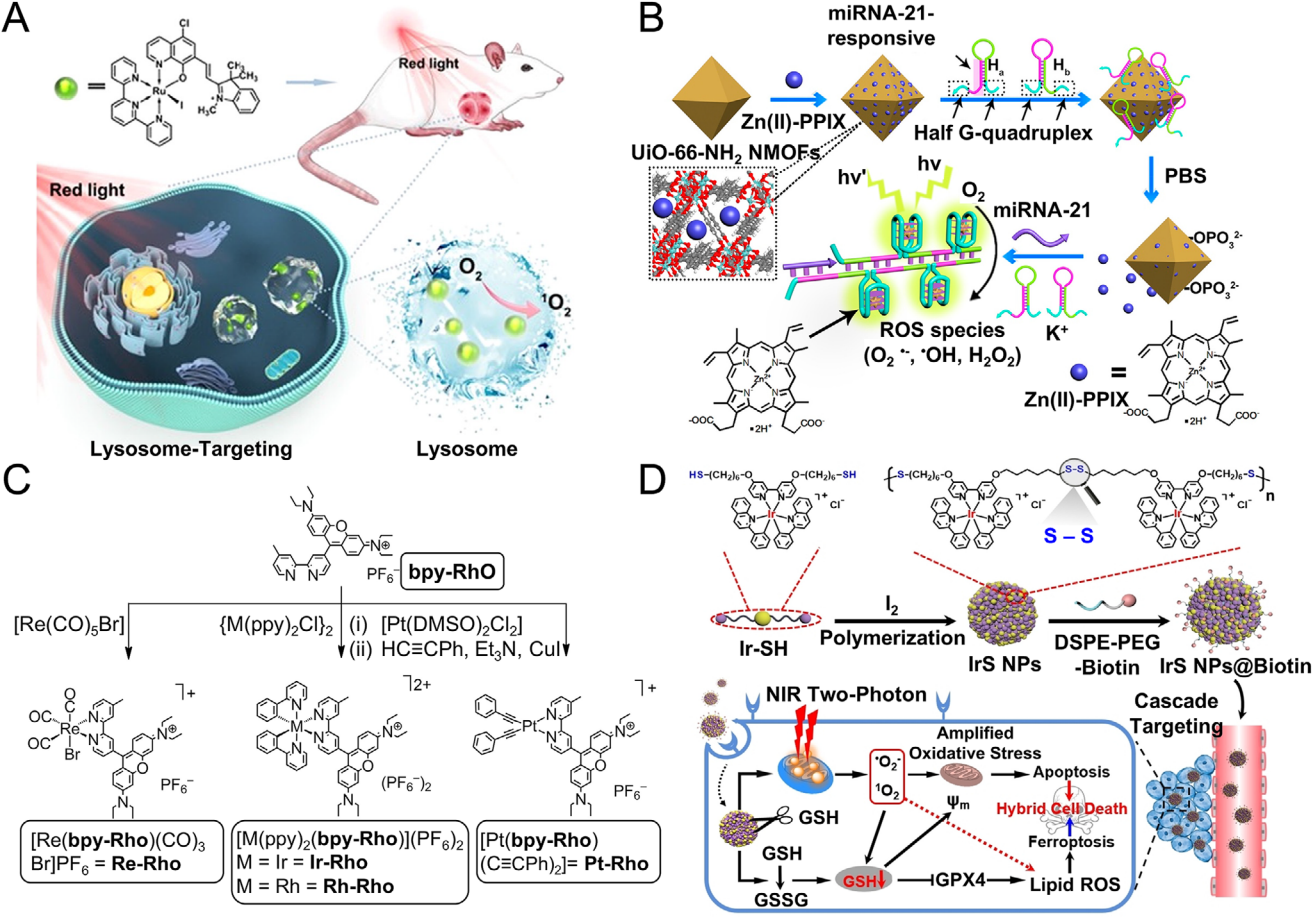
Representative studies utilizing metal‐ligand complexes as photosensitizers for PDT. A) Depiction of lysosome‐targeting Ru complexes with large‐conjugated indoline‐pyridobenzopyrans for PTT. Reproduced with permission.^[^ ^85^ ^]^ Copyright 2022, American Chemical Society. B) Schematic of NMOFs incorporating the Zn(II)‐PPIX photosensitizer and DNA hairpins. Reproduced with permission.^[^ ^86^ ^]^ Copyright 2022, American Chemical Society. C) Chemical structures of rhodamine‐functionalized transition metal complexes (M‐Rho) serving as photosensitizers for PDT. Reproduced with permission.^[^ ^89^ ^]^ Copyright 2019, American Chemical Society. D) Illustration of the preparation and therapeutic mechanism of the Ir(III) coordination polymer through ferroptosis and PDT‐mediated apoptosis. Reproduced with permission.^[^ ^94^ ^]^ Copyright 2022, Wiley.

Ru(II) complexes have emerged as promising photosensitizers due to their high water solubility, excellent photostability, and efficient ROS generation capabilities. These complexes typically rely on UV or short‐wavelength visible light to generate ROS. However, for treating deep‐seated or large tumors, the development of Ru(II) complexes that respond to red or NIR light within the therapeutic window (650–900 nm) is essential. He et al. (2021) synthesized Ru complexes (Ru‐I) using large‐conjugated indoline‐pyridobenzopyrans as ligands (**Figure** **5**A).^[^ ^85^ ^]^ This resulted in a 100 nm red shift in absorption compared to conventional Ru polypyridine complexes, with a prominent absorption band at 660 nm. Ru‐I complexes targeted lysosomes and disrupted their structure and function under red‐light irradiation, leading to cancer cell death and significant tumor growth inhibition in murine tumor models.

In another study, nucleic acid‐modified UiO‐66‐NH2 MOFs (NMOFs) were designed to load Zn(II)‐protoporphyrin IX (Zn(II)‐PPIX) as a photosensitizer for miRNA‐guided imaging and PDT (Figure 5B).^[^ ^86^ ^]^ The system utilized Zr^4+^ ions within the NMOFs to bind two types of hairpin DNA backbones containing miRNA recognition sequences (miRNA‐21 or miRNA‐221) and G‐quadruplex units. Phosphate ions within cells replaced the hairpin DNA, releasing Zn(II)‐PPIX and initiating a hybridization chain reaction upon interaction with the target miRNA. This process resulted in the assembly of G‐quadruplex chains loaded with fluorescent Zn(II)‐PPIX, enabling selective miRNA‐guided imaging of cancer cells and ROS‐mediated tumor cell destruction upon visible light irradiation.

Ir(III) complexes are also notable photosensitizers due to their excellent water solubility, strong singlet oxygengeneration, long phosphorescence lifetimes, and two‐photon absorption characteristics. Recent developments include donor–acceptor (D–A) structured Ir(III) complexes with auxiliary fluorophore ligands for enhanced ROS generation and photodynamic efficacy.^[^ ^87^ ^]^ These D‐A coordinated Ir(III) complexes demonstrated efficient energy transfer from MLCT states to triplet states, facilitating ROS production. For targeted delivery, M1 phenotype macrophage‐derived exosomes (M1‐Exos) were conjugated with D‐A coordinated Ir(III) complexes to form M1‐Exo/Ir nanoparticles. These nanoparticles exhibited tumor accumulation and significant antitumor effects in 4T1 tumor‐bearing mice upon white light irradiation.

Efforts to expand the absorption range of photosensitizers within the phototherapeutic window (600–900 nm) include conjugating organic fluorophores with metal complexes. For example, coumarin‐pyridine fluorophores conjugated with cyclometalated Ru(II) polypyridyl complexes exhibited red‐light absorption and generated ROS to induce cancer cell death in HT‐29, CT‐26, HeLa, and A2780 cell lines.^[^ ^88^ ^]^ Similarly, rhodamine‐tethered bipyridine ligands chelated with Re(I), Ir(III), Rh(III), and Pt(II) metals demonstrated tumor‐selective ROS production and phototoxicity. Among these, Ir(III) complexes showed the highest ROS generation and antitumor efficacy due to efficient intersystem crossing and spin‐orbit coupling, as confirmed in MCF‐7 tumor‐bearing mice.^[^ ^89^ ^]^ The rhodamine‐tethered bipyridine ligands were particularly effective in promoting selective ROS generation due to their unique ability to transfer energy efficiently between the singlet state of rhodamine and the triplet state of the transition‐metal complex (Figure 5C). This transition enables enhanced ROS production from the rhodamine triplet state, which accumulates preferentially in the mitochondria of tumor cells due to their higher negative charge compared to normal cells. This selective accumulation facilitates ROS‐induced apoptosis in cancer cells. Among the metals studied, Ir(III) complexes exhibited superior ROS generation and cytotoxicity, attributed to effective intersystem crossing and spin‐orbit coupling mechanisms. These properties translated into significant tumor accumulation and antitumor efficacy in an MCF‐7 tumor‐bearing mouse model, demonstrating their potential as efficient PDT agents.

Another innovative approach involved combining fluorophores with metal complexes to develop photosensitizers that also serve as imaging agents. Acetylacetonated boron‐dipyrromethene (BODIPY) conjugated with biscyclometalated Ir(III) complexes was developed as a fluorescent‐PDT agent.^[^ ^90^ ^]^ This agent achieved a balance between fluorescence emission and singlet oxygen production under visible light irradiation. Fluorescence emission originated from the BODIPY singlet state, while efficient intersystem crossing to the BODIPY triplet state, facilitated by MLCT interactions, led to ROS generation. These dual functionalities enabled imaging and therapeutic applications in HeLa cells, combining diagnostic capability with high phototoxicity.

To address the hypoxic conditions of the TME, a catalytic PDT system was developed using upconversion nanoparticles (UCNPs) as a core and zirconium/iron porphyrin MOFs as a shell, with ultrasmall gold nanoparticles (Au NPs) as artificial enzymes.^[^ ^91^ ^]^ In this system, Au NPs acted like glucose oxidase, converting glucose into hydrogen peroxide, while the iron porphyrin MOF decomposed hydrogen peroxide into oxygen, continuously supplying oxygen within the hypoxic tumor. Under NIR irradiation, UCNPs absorbed light and transferred energy via fluorescence resonance energy transfer (FRET) to the MOFs, exciting the porphyrin cores to generate ROS. This system significantly increased oxygen levels in tumors and demonstrated tumor suppression and eradication in U87MG tumor‐bearing mice.

Recently, Repetowski et al. (2024) reported the development of novel metal‐ligand complexes composed of tert‐butylsulfonyl‐substituted phthalocyanine ligands coordinated with metal centers such as Zn(II) or Pt(II) for enhanced NIR‐mediated PDT.^[^ ^92^ ^]^ To overcome the inherent issues of self‐aggregation associated with phthalocyanine‐based complexes and to improve their bioavailability and tumor specificity, these metal‐ligand complexes were encapsulated within Pluronic P123 polymeric micelles. Encapsulation not only prevented aggregation but also substantially improved the stability, solubility, and targeted delivery of these complexes to tumor tissues. The Pt(II)‐based complex exhibited exceptional singlet oxygen generation, whereas the Zn(II)‐based complex demonstrated broader ROS generation, including both singlet oxygen and highly reactive hydroxyl radicals. Significantly, these encapsulated metal‐ligand complexes demonstrated potent antitumor activities not only against conventional colorectal cancer cells and mouse xenograft tumor models but also against advanced, human‐induced pluripotent stem cell‐derived colonic organoids, an advanced ex vivo human cancer model. Notably, when employed in vascular‐targeted PDT, characterized by short drug‐to‐light intervals and selective targeting of tumor vasculature, the Zn(II) complex achieved complete tumor eradication in 84% of treated cases, whereas the Pt(II) complex impressively reached 100% tumor clearance. These results demonstrated the tremendous therapeutic potential of rational ligand design and optimized metal coordination in enhancing the efficacy and clinical applicability of metal‐ligand complexes for advanced PDT modalities.

#### 5.1.2 Metal‐Ligand Complex‐based Photosensitizers Combined with Other Therapeutic Strategies

As mentioned, the intrinsic or acquired resistance of cancer cells to apoptosis can limit PDT effectiveness. To overcome this limitation, recent research has focused on integrating additional RCD pathways, such as ferroptosis and pyroptosis, into PDT‐based strategies. Ferroptosis is a non‐apoptotic form of RCD characterized by iron‐dependent lipid peroxidation that damages cellular membranes. This pathway selectively targets cancer cells with dysregulated iron metabolism or antioxidant systems, minimizing harm to normal cells.^[^ ^96^ ^]^ Ferroptosis has shown efficacy in drug‐resistant tumors with fewer systemic side effects and synergistic potential when combined with existing therapies.^[^ ^97^ ^]^ In particular, the ROS generated during PDT can directly attack tumor cells, accelerate lipid peroxidation, and improve the hypoxic TME, thereby enhancing the therapeutic efficacy of PDT.^[^ ^98^ ^]^

Feng et al. (2023) developed a synergistic PDT system combined with ferroptosis to treat melanoma, a skin cancer that often exhibits resistance to conventional PDT due to melanin's strong absorption in the 500–600 nm range.^[^ ^93^ ^]^ To overcome this limitation, the researchers utilized two‐photon absorption, enabling activation at NIR wavelengths (700–1000 nm) where melanin absorption is minimal. Ir(III) complexes modified with polyphenols capable of coordinating Fe^3+^ ions were self‐assembled into nanopolymers (Ir‐Fe NPs). These NPs dissociate in the acidic lysosomal environment into Ir(III) complexes and Fe^3+^ ions. Upon two‐photon irradiation, the Ir(III) complexes produced ROS, inducing apoptosis and autophagy, while Fe^3+^ ions contributed to ferroptosis by depleting GSH, generating lipid peroxides, and reducing GPX4. Encapsulation of Ir‐Fe NPs within A375 melanoma‐derived EXOs enhanced tumor targeting, resulting in significant tumor accumulation and near‐complete tumor eradication in murine melanoma models after a single treatment.

Ke et al. (2022) synthesized coordination polymeric nanoparticles (IrS NPs) through the oxidative polymerization of thiol‐functionalized Ir(III) complexes (Figure 5D).^[^ ^94^ ^]^ These nanoparticles are responsive to the intracellular redox environment, breaking disulfide bonds in the presence of GSH to release monomeric Ir(III) complexes. This release reduces intracellular GSH levels, thereby enhancing the therapeutic effects of ROS. Accumulating primarily in mitochondria, IrS NPs generated a mixture of singlet oxygen and superoxide anions, inducing mitochondrial oxidative stress. Additionally, lipid peroxidation mediated by ferroptosis amplified cell death through a combination of apoptosis and ferroptosis. To improve tumor selectivity, IrS NPs were encapsulated in amphiphilic biotin‐functionalized polymers. In an A549 tumor‐bearing mouse model, these nanoparticles achieved high tumor accumulation and near‐complete tumor elimination following a single two‐photon irradiation session.

Li et al. (2024) reported a sophisticated photo‐activated therapeutic platform utilizing an Fe‐based MOF catalyst with a unique Fe–O–Cu bridging structure, assembled through coordination with Cu^2+^ and the electron‐rich ligand gallic acid.^[^ ^95^ ^]^ Leveraging gallic acid's robust electron‐donating capacity, this specialized photosensitizer effectively suppressed the recombination of electron‐hole pairs, thus significantly enhancing the electron‐transfer efficiency required for PDT under visible‐light irradiation. Additionally, cinnamaldehyde was integrated into the structure to continuously supply H_2_O_2_, further promoting ROS generation, while hyaluronic acid (HA) functionalization provided active tumor‐targeting capabilities. Upon irradiation with a 650 nm laser, the MOF photosensitizer demonstrated efficient ROS production, resulting in potent PDT effects. Moreover, this structure utilized the TME's GSH‐rich conditions to catalytically reduce Cu^2+^ ions to Cu^+^. The resulting Cu^+^ ions notably accelerated the Fe^3+^/Fe^2+^ redox cycling within the MOF, effectively mimicking peroxidase‐like activity to significantly enhance ROS generation via CDT. Furthermore, the coordinated Fe ions released during the therapeutic process served as MRI contrast agents, enabling precise real‐time tumor monitoring and treatment evaluation. This comprehensive integration of PDT, CDT, ferroptosis induction, and diagnostic imaging enables the multifunctional and synergistic therapeutic effect of this metal‐ligand‐based nanoplatform, substantially advancing its application for highly effective multimodal cancer therapy.

### 5.2 Metal‐Ligand Complexes as a Carrier of Photosensitizers for PDT

Challenges such as poor water solubility, low cellular uptake, and insufficient tumor targeting have prompted the development of multifunctional nanomaterials to improve PDT agent delivery. These include vesicles, micelles, inorganic nanoparticles, and viral nanoparticles, all enhancing delivery efficiency.^[^ ^99^ ^]^ Among them, MOFs and MPNs stand out as effective carriers. MOFs offer high surface area, biocompatibility, and tunable structures that can encapsulate photosensitizers or incorporate them as ligands, preventing self‐aggregation and enhancing photodynamic activity. Additionally, MOFs can carry functional agents like oxygen releasers or ROS modulators to improve the TME and boost PDT efficacy. MPNs, easily formed via coordination‐driven self‐assembly, benefit from the strong adhesive properties of polyphenols, allowing them to coat substrates of diverse shapes and morphologies.^[^ ^100^, ^101^ ^]^ Additionally, MPNs can be functionalized for targeted and stimuli‐responsive drug release, such as pH‐triggered delivery within acidic TMEs. These metal‐ligand complexes improve photosensitizer solubility, enable controlled release, and enhance tumor targeting, overcoming challenges in traditional photosensitizer delivery. **Table** **3** summarizes recent studies employing various systems, including MOFs and MPNs, for photosensitizer delivery.

**Table 3:** Overview of metal‐ligand complexes as carriers in PDT and complementary cancer therapies: summarizing therapy types, metal ions, ligands, therapeutic agents, platform sizes, tumor models, target diseases, and light sources for activation.

Therapy type	Metal ion	Ligand	Therapeutic agents	Size	Tumor model	Disease	Light source	References
PDT	Ru(III), Pt(IV), Zr(IV)	Bipyridine ligands	Ce6 as a photosensitizer	125 nm	H22 tumor‐bearing mice	Cancer	660 nm	[102]
PDT	Zr(IV)	Carboxyl‐ate‐functional‐ized BODIPY ligand	BODIPY as a photosensitizer	171 nm	Triple‐negative luciferase‐expressing MDA‐MB‐231 tumor‐bearing mice	Cancer	525 nm	[103]
PDT	Zr(IV)	1, 4‐benzene dicarboxy‐late	ICG as a photosensitizer	91.2 nm	MCF‐7 tumor‐bearing mice	Cancer	808 nm	[104]
PDT	Zn(II)	Imidazol	UCNP and MB as photosensitizers	∼500 nm	PL 45 cells	Cancer	980 nm	[105]
PDT	Fe(III)	Folic acid‐PEG‐phenol, PEG‐polyphenol	HMME as a photosensitizer	2.3 ± 0.3 µm	HeLa cells	Cancer	638 nm	[106]
PDT	Fe(III)	TA	MB as a photosensitizer, SRF and Fe(III) as ferroptosis inducers	220 nm	4T1 tumor‐bearing mice	Cancer	660 nm	[107]
PDT, chemothe‐rapy	Cu(II)	Carboxylate	TBD as a photosensitizer, Pt(IV) complex serving as both a hypoxia modulator and a Pt(II) chemotherapy prodrug	∼160 nm	4T1 tumor‐bearing mice	Cancer	400–700 nm	[108]
PDT, chemothe‐rapy, immunoth‐erapy	Fe(II)	Gossypol, PEG‐Ce6 polyphenol	Ce6 as a photosensitizer, gossypol as a drug	32.7 nm	4T1 tumor‐bearing mice	Cancer	660 nm	[109]
PDT, immunoth‐erapy	Mn(II)	Polyphenol, dsDNA (cGAS‐STING pathway)	Ce6 as a photosensitizer, Mn(II) as a cGAS‐STING agonist	∼200 nm	4T1 tumor‐bearing mice	Cancer	660 nm	[110]
PDT, immunoth‐erapy	Zn(II)	Imidazol	IR780 as a photosensitizer, Zn(II) as a cGAS‐STING agonist, AQ4N as a hypoxic drug	190.2 nm	4T1 tumor‐bearing mice	Cancer	NIR	[111]
PDT, immunoth‐erapy	Zr(IV)	1, 4‐bezenedic‐arboxylic acid	Meso‐tetra(carboxy‐phenyl)porphy‐rin as a photosensitizer, SR‐717 as a STING agonist	51.2 nm	4T1 tumor‐bearing mice, 4T1 cells	Cancer	650 nm	[112]
PDT, CDT, chemothe‐rapy	Fe(II)	Hematop‐orphyrin monomethyl ether and thalidomide	Hematoporph‐yrin monomethyl‐ether as a photosensitizer, thalidomide as an anti‐angiogenesis drug	60 nm	4T1 tumor‐bearing mice, 4T1 cells	Cancer	808 nm	[113]

#### 5.2.1 Strategies for Tumor Microenvironment Modulation in Photodynamic Therapy: Progress in Metal‐Ligand Complexes and Nanomaterials

The TME, characterized by an acidic pH, excessive production of adenosine triphosphate (ATP), hydrogen peroxide, and antioxidants, hypoxia, and low catalase activity, provides an ideal setting for tumor growth, proliferation, and metastasis.^[^ ^114^ ^]^ However, these features also significantly impede effective cancer therapies. From a PDT perspective, hypoxia in tumors activates antioxidant enzymes, increases intracellular GSH, and strengthens antioxidant defenses, thereby reducing ROS levels. Furthermore, the oxygen dependency of ROS generation diminishes PDT efficiency in hypoxic tumors. To address these challenges, various strategies have been developed to modulate the TME and enhance PDT outcomes.

Pan et al. (2023) designed a catalase‐ and GSH oxidase‐mimicking nanozyme (ZMRP) by incorporating Ru^3+^ and Pt^4+^ into a UiO‐67 MOF.^[^ ^102^ ^]^ This Zr^4+^‐MOF‐Ru^3+^/Pt^4+^ (ZMRP) structure, loaded with chlorin e6 (Ce6) as a photosensitizer, formed a composite system (ZMRPC) for PDT. In hypoxic tumor environments, ZMRPC catalyzed the continuous decomposition of hydrogen peroxide to generate oxygen, enhancing PDT efficacy. Additionally, it depleted GSH to weaken antioxidant defenses, further amplifying ROS‐mediated cytotoxicity. To improve tumor targeting and biocompatibility, ZMRPC was modified with HA to create ZMRPC@HA, which effectively suppressed tumor growth and proliferation under light irradiation.

Chen et al. (2024) reported a BODIPY‐based Zr‐MOF system combined with fluorinated phosphate‐functionalized methoxy PEG (F‐PEG).^[^ ^103^ ^]^ The perfluorooctyl groups within F‐PEG facilitated oxygen delivery to hypoxic tumors, alleviating oxygen deficiency. The BODIPY component acted as a photosensitizer, generating ROS under LED irradiation, while the system's hydrogel formulation prolonged retention at tumor sites. In a triple‐negative breast cancer model (MDA‐MB‐231), this approach demonstrated effective tumor growth suppression and size reduction.

**Figure 6:**
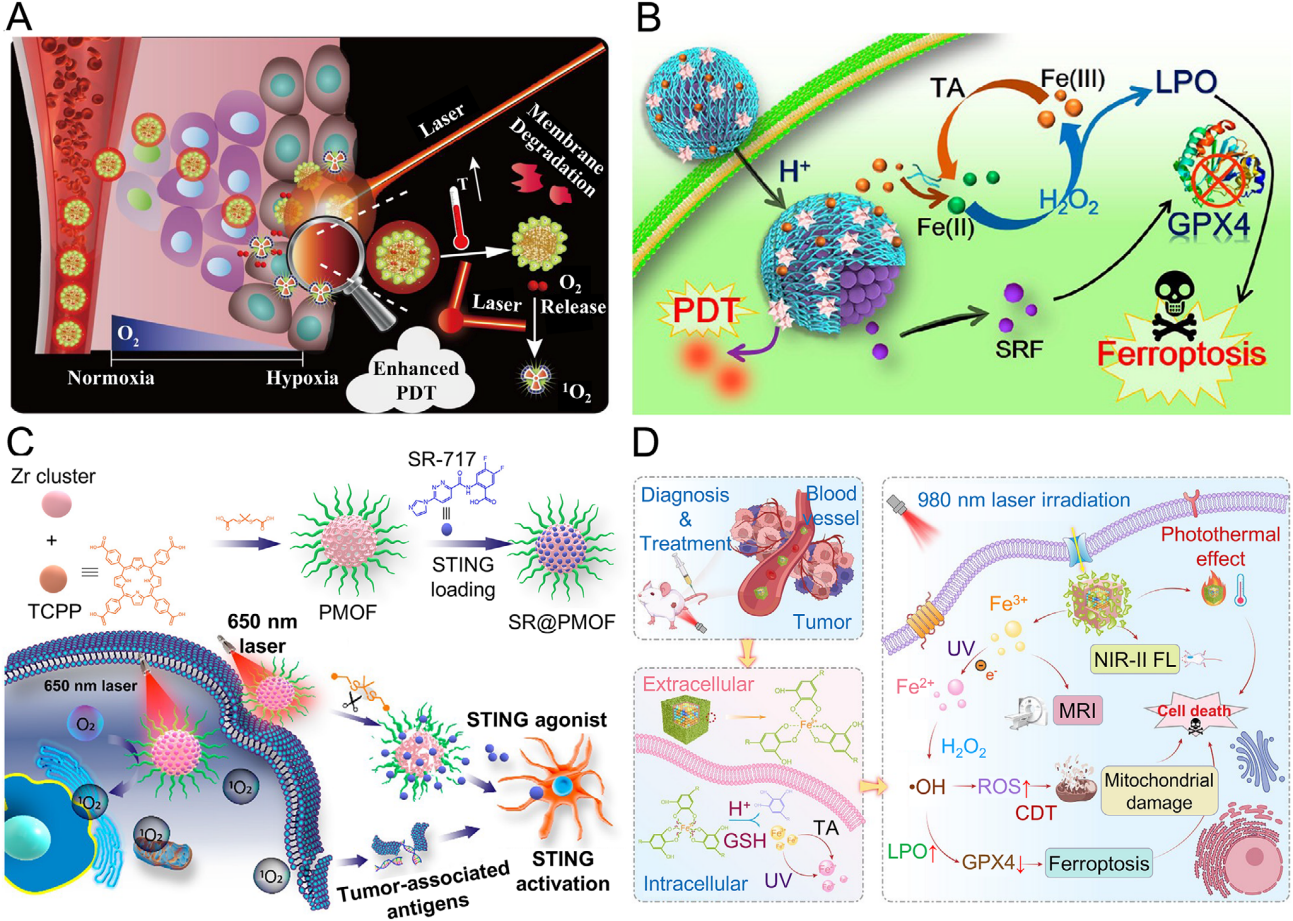
Representative studies employing metal‐ligand complexes as carriers for PDT. A) Schematic of the storing and releasing oxygen mechanism of O_2_@UiO‐66@ICG@RBC nanoparticles. Reproduced with permission.^[^ ^104^ ^]^ Copyright 2018, Elsevier B) Illustration of an MPN‐based system designed to trigger PDT while concurrently inducing ferroptosis through iron redox cycling. Reproduced with permission.^[^ ^107^ ^]^ Copyright 2018, American Chemical Society. C) Illustration of the preparation and therapeutic mechanism of PMOF NPs with STING agonist SR‐717. Reproduced with permission.^[^ ^112^ ^]^ Copyright 2023, American Chemical Society. D) Depiction of multiple anticancer mechanisms of coordination‐driven nanoparticles composed of Er^3+^, Ce^3+^, and Tm^3+^ co‐doped core‐shell nanoparticles and Fe‐TA complex. Reproduced with permission.^[^ ^78^ ^]^ Copyright 2024, Wiley.

Gao et al. (2018) developed a UiO‐66‐based system incorporating indocyanine green (ICG) as an NIR photosensitizer. The surface of UiO‐66 was modified with sulfonic acid for ICG loading through coordination with Zr_6_ clusters (**Figure** **6**A). To address hypoxia, the nanoparticles were coated with red blood cell (RBC) membranes, forming O_2_@UiO‐66@ICG@RBC nanoparticles capable of storing and releasing oxygen.^[^ ^104^ ^]^ Due to the immunomodulatory proteins of RBC membranes, the system can evade recognition by the immune system in vivo, allowing for efficient circulation and passive tumor targeting through the enhanced permeability and retention effect. Upon 808 nm laser irradiation, ICG generated singlet oxygen, breaking down the RBC membranes and releasing stored oxygen, which enhanced PDT efficacy. This system exhibited significant antitumor effects in MCF‐7 tumor spheroids and xenograft models.

Cai et al. (2017) introduced a nanocomposite combining UCNPs with methylene blue (MB) as a core, coated with zeolitic imidazolate framework‐8 (ZIF‐8) and functionalized with catalase.^[^ ^105^ ^]^ Upon 980 nm irradiation, energy transfer from UCNPs to MB via FRET triggered the generation of singlet oxygen. Simultaneously, catalase converted endogenous hydrogen peroxide into oxygen, increasing PDT efficiency and promoting cancer cell apoptosis.

Wei et al. (2018) developed PEG‐MPN capsules loaded with haematoporphyrin monomethyl ether (HMME) as a PS (MPN@HMME).^[^ ^106^ ^]^ By modifying PEG with folic acid, the capsules targeted folate receptors on cancer cells, facilitating uptake. In the acidic lysosomal environment, the MPN capsules disassembled, releasing the photosensitizer into the cytoplasm. Upon 638 nm laser irradiation, the ROS generated by HMME effectively induced apoptosis in HeLa cells.

#### 5.2.2 Metal‐Ligand Complex‐Based Carriers Combined with Other Therapeutic Strategies

To overcome the limitations of apoptosis, an MPN‐based system that provides and regenerates iron ions for simultaneous ferroptosis and PDT‐mediated apoptosis has been developed (Figure 6B).^[^ ^107^ ^]^ This system uses SRF, a ferroptosis inducer, as the core, coated with an MPN shell formed by Fe^3+^ ions and TA, and loaded with methylene blue as both a PDT and fluorescent imaging agent. The MPN shell disassembles under lysosomal acidic conditions, releasing Fe^3+^ ions and SRF to initiate ferroptosis. Released Fe^3+^ ions or those generated via ferroptosis chelate with TA under neutral pH and are reduced to Fe^2^⁺, which further accelerates lipid hydroperoxide generation through Fenton‐like reactions. The system effectively induced ferroptosis and cytotoxicity against cancer cells under 660 nm light irradiation, achieving antitumor effects in a 4T1 tumor‐bearing mouse model. However, the potential light absorption by the Fe^3+^‐TA complex at 660 nm and its influence on PDT efficiency remain unexplored.

Wang et al. (2020) reported a Cu‐based MOF for improving hypoxic conditions and combining PDT with chemotherapy.^[^ ^108^ ^]^ The Cu(II) carboxylate‐based MOF (MOF‐199) was loaded with a Pt(IV)‐diazido complex and encapsulated with tetraphenylbutadiene (TBD)‐PEG, a PEG‐modified aggregation‐induced‐emission photosensitizer. Upon exposure to high GSH levels in the TME, MOF‐199 decomposed, releasing TBD and the Pt complex. Under visible light (400–700 nm) irradiation, the Pt(IV) complex was reduced to Pt(II), generating oxygen to mitigate hypoxia and acting as a chemodrug. Concurrently, TBD generated ROS and emitted bright fluorescence, enabling imaging‐guided therapy. This system effectively suppressed tumor growth in a 4T1 tumor‐bearing mouse model.

ICD offers a promising approach to addressing tumor metastasis and recurrence by inducing antitumor immune responses. During PDT, cancer cell death promotes the extracellular release of DAMPs such as calreticulin protein, high mobility group box 1, and ATP, along with the exposure of tumor‐associated antigens.^[^ ^115^ ^]^ These signals stimulate the maturation of antigen‐presenting cells, such as DCs, and activate T lymphocytes through inflammatory cytokine production, inducing immune‐mediated tumor eradication.^[^ ^116^ ^]^ Combining PDT with chemotherapy further enhances ICD stimulation. Zhang et al. (2021) designed PEG‐Ce6‐Fe^2+^‐gossypol MPNs) to synergistically induce PDT, chemotherapy, and immunotherapy.^[^ ^109^ ^]^ These MPNs incorporated gossypol, a polyphenolic compound with anticancer properties, and a PEG‐Ce6 polyphenol conjugate via Fe^2+^‐mediated coordination. Under 660 nm laser irradiation, PEG‐Ce6‐Fe^2+^‐gossypol MPNs effectively induced cancer cell death in a 4T1 tumor‐bearing mouse model through PDT and chemotherapy while stimulating the immune system. The system promoted DC maturation, cytokine production, and cytotoxic T lymphocyte (CTL) infiltration. The addition of PD‐L1 antibodies further enhanced immune stimulation, reducing tumor growth and metastasis.

Tian et al. (2024) developed a mitochondria‐targeting metal‐phenolic nanomaterial (PFM) to activate the cyclic GMP‐AMP synthase‐stimulator of interferon genes (cGAS‐STING) pathway and induce antitumor immunity.^[^ ^110^ ^]^ The PFM system utilized an amphiphilic phenolic polymer containing polyphenols, triphenylphosphonium, Ce6, and furan moieties, which self‐assembled with Mn^2+^ through metal‐phenolic coordination. Under 660 nm light irradiation, the system generated ROS that oxidized furan units in mitochondria, causing mitochondrial DNA (mtDNA) damage and crosslinking. This led to the release of mtDNA fragments, which were absorbed by tumor‐associated macrophages (TAMs). The combination of Mn^2+^ and tumor‐derived mtDNA activated the cGAS‐STING pathway, reprogramming M2‐like TAMs to an M1 phenotype and inducing T cell‐mediated antitumor immunity. Antitumor efficacy was demonstrated in a 4T1 tumor‐bearing mouse model, with cGAS‐STING activation shown to enhance T cell‐mediated immunity.

Guo et al. (2024) developed a MOF‐based nanoagonist for NIR‐responsive degradation and TME modulation, combining PDT and immunotherapy.^[^ ^111^ ^]^ The nanoagonist incorporated IR780, a photosensitizer, and banoxantrone dihydrochloride (AQ4N), a hypoxia‐activated prodrug, into polydopamine‐modified ZIF‐8. Upon NIR irradiation, the system generated mitochondria‐targeting ROS, leading to oxidative damage and increased mtDNA release. The polydopamine coating depleted intracellular GSH, enhancing PDT effects, while AQ4N inhibited nuclear DNA repair. Released mtDNA acted as a DAMP to activate the cGAS‐STING pathway, promoting DC maturation and CTL infiltration. This nanoagonist demonstrated potent antitumor immunity, enabling primary tumor eradication and long‐term suppression of metastasis.

Recently, Zhou et al. (2023) reported polymeric MOF nanoparticles (PMOF NPs) specifically designed to combine PDT with immunotherapy via STING pathway activation (Figure 6C).^[^ ^112^ ^]^ These PMOF nanoparticles were synthesized using Zr^4+^ coordinated with a block copolymer ligand consisting of PEG and polyacrylamide bearing 1, 4‐benzenedicarboxylic acid units, meso‐tetra(carboxyphenyl)porphyrin as a photosensitizer, and thioketal diacetic acid linkers. The resulting nanoparticles exhibited a core‐shell structure with excellent physiological stability due to their PEG outer shell. Further, the porous nature of the PMOF allowed efficient encapsulation of the potent STING agonist SR‐717, generating multifunctional SR@PMOF nanoparticles. Upon irradiation at 650 nm, the meso‐tetra(carboxyphenyl)porphyrin effectively produced singlet oxygen, triggering oxidative stress‐induced tumor cell apoptosis. Simultaneously, oxidative cleavage of the thioketal linkages within the nanoparticles led to rapid and selective release of the encapsulated SR‐717, thus significantly amplifying immunotherapeutic effects through activation of the STING signaling pathway. In vivo studies using a 4T1 breast tumor mouse model demonstrated that this combined PDT and immune activation strategy effectively inhibited both primary tumor growth and secondary metastatic tumor formation, highlighting the promising therapeutic potential of such metal‐ligand‐based multifunctional systems for integrated cancer treatment approaches.

Additionally, Qu et al. (2025) recently developed sophisticated nanoplatforms via coordination‐driven self‐assembly of hematoporphyrin monomethyl ether (a PDT photosensitizer), Fe^2+^, and the anti‐angiogenic drug thalidomide, integrating multiple anticancer mechanisms including PDT, CDT, chemotherapy, and ferroptosis (Figure 6D).^[^ ^113^ ^]^ Upon NIR irradiation, the hematoporphyrin moiety generated ROS for effective PDT. Concurrently, under the acidic TME, these nanoparticles underwent controlled degradation, releasing both thalidomide and Fe^2+^ directly into tumor tissue. The released thalidomide initiated anti‐angiogenic chemotherapy, inhibiting tumor vasculature, while Fe^2+^ participated in CDT by catalyzing the Fenton reaction, converting tumor‐overexpressed hydrogen peroxide into highly cytotoxic hydroxyl radicals. Furthermore, the oxidized iron ions (Fe^3+^) depleted intracellular GSH, inducing lipid peroxidation and triggering ferroptosis. Fe^2+^ also directly contributed to ferroptosis through lipid peroxidation mechanisms, further amplifying therapeutic efficacy. Consequently, the synergistic interplay among ROS generation, chemotherapy‐induced stress, GSH depletion, and ferroptosis resulted in maximized oxidative stress within cancer cells, effectively leading to apoptotic cell death. Additionally, Fe^2+^ ions served as MRI contrast agents, offering the advantage of precise, real‐time imaging to optimize therapeutic timing and monitor treatment response. Collectively, this multifunctional nanoparticle design demonstrates a significant step forward in multimodal cancer therapy, combining imaging and diverse therapeutic strategies in one unified platform.

## 6 Photo‐Activated Chemotherapy

PACT is a treatment approach where inactive, non‐toxic prodrugs are selectively activated at tumor sites by targeted light exposure. This activation causes structural changes in metal‐ligand complexes, often through metal‐ligand bond cleavage or metal center photoreduction, releasing cytotoxic metal species or ligands that kill cancer cells.^[^ ^117^ ^]^ In contrast to PDT, PACT functions independently of oxygen, thereby effectively addressing PDT's primary limitations, such as oxygen dependence and reduced efficacy in hypoxic TMEs. However, PACT currently remains in the early stages of development, necessitating active and ongoing research efforts. In this section, we discuss recent prominent examples of the design, mechanism, and therapeutic outcomes of metal‐ligand complexes as promising PACT drug candidates. **Table** **4** summarizes representative systems, describing their therapeutic characteristics, composition, tumor models, and photochemical activation parameters.

**Table 4:** Overview of metal‐ligand complexes in PACT and complementary cancer therapies: summarizing therapy types, metal ions, ligands, therapeutic agents, platform sizes, tumor models, target diseases, and light sources for activation.

Therapy type	Metal ion	Ligand	Role of metal‐ligand complex	Size	Tumor model	Disease	Light source	References
PACT	Au(III)	2, 6‐diphenyl‐pyridine, alkyl	PACT drug	160 nm	Zebrafish, A375‐bearing mice, HCT116, and MDA‐MB‐231 cells	Cancer	420 nm	[118]
PACT	Au(III)	2, 6‐diphenyl‐pyridine, hydride	PACT drug	‐	Zebrafish, HepG2 cells	Cancer	420 nm	[119]
PACT	Au(III)	2, 6‐diphenyl‐pyridine, hydride	PACT drug	‐	Zebrafish, H3122 cells	Cancer	600 nm, 630 nm	[120]
PACT	Pt(IV)	Oxaliplatin, coumarin derivative, succinic acid	PACT drug	‐	A549cisR cells	Cancer	450 nm	[121]
PACT	Pt(IV)	Oxaliplatin, coumarin derivative, dimethylx‐anthenone‐4‐acetic acid	PACT drug	‐	4T1 tumor‐bearing mice, lung cancer stem cells	Cancer	880 nm	[122]
PACT	Ru(II)	Polypyridyl	PACT drug	‐	HEK T‐Rex cells	Cancer	660 nm	[123]

### 6.1 Gold‐Based Complexes (Au(I) and Au(III))

Au complexes have gained significant interest as potent anticancer agents due to their specific targeting of thiol‐ or selenol‐containing enzymes and proteins, such as thioredoxin reductase, which are critical to tumor cell survival and proliferation. Moreover, Au complexes have demonstrated efficacy against cisplatin‐resistant cancer cell lines.^[^ ^124^ ^]^ However, the high reactivity of Au complexes can result in undesirable non‐specific reactions with thiol‐containing biomolecules in normal cells or serum proteins, reducing therapeutic effectiveness and increasing systemic toxicity.^[^ ^125^ ^]^ To overcome these limitations, recent studies have strategically designed Au complexes as PACT agents to enhance selective cytotoxicity.

**Figure 7:**
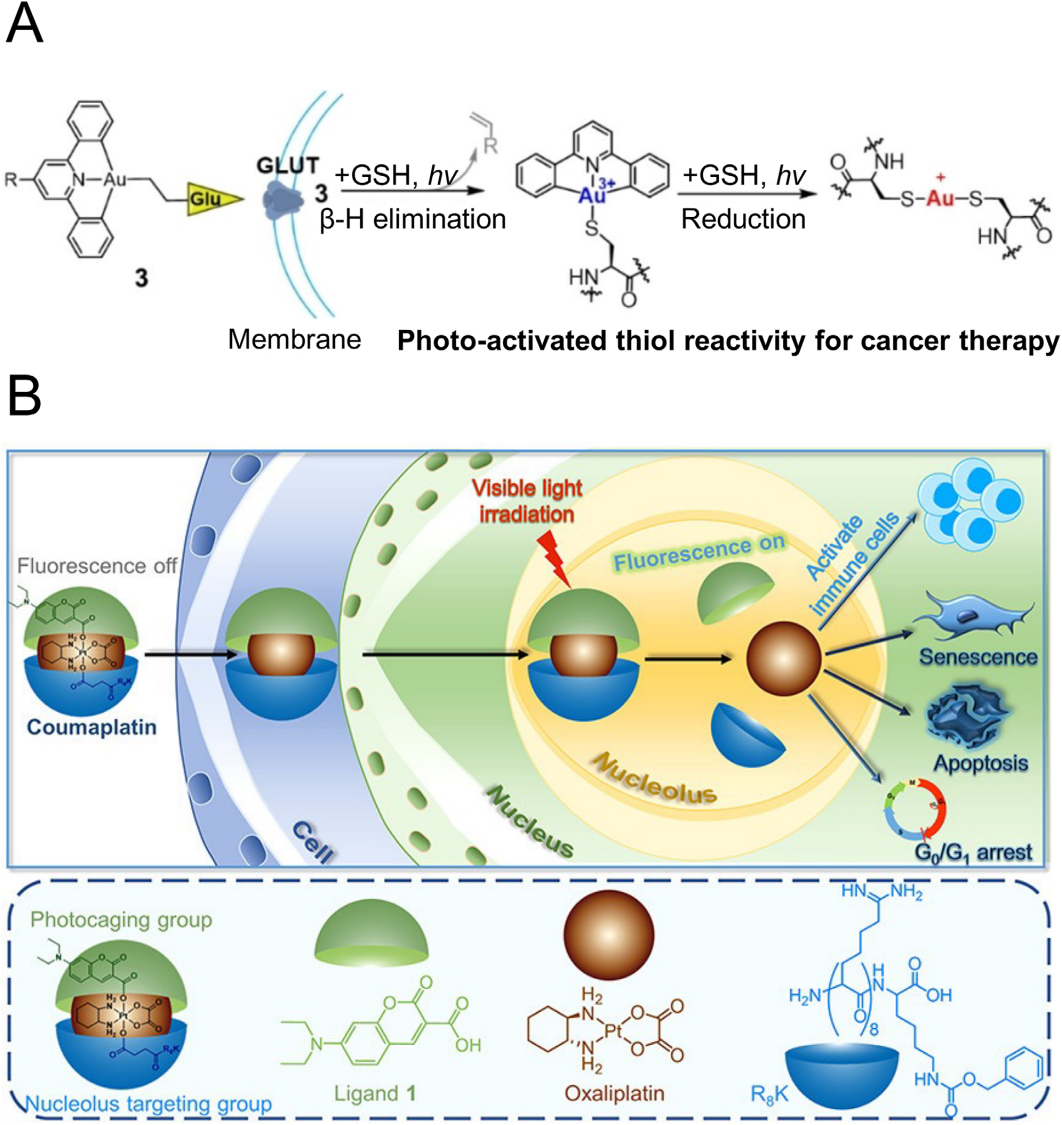
Representative studies on metal‐ligand complexes for PACT. A) Illustration of the photoreactivity of alkylgold(III) complexes. Reproduced with permission.^[^ ^118^ ^]^ Copyright 2022, Wiley. B) Illustration of visible light‐activation of Pt(IV) oxaliplatin complexes conjugated with coumarin‐based ligands. Reproduced with permission.^[^ ^121^ ^]^ Copyright 2020, American Chemical Society.

For instance, Jiang et al. (2022) developed glucose‐functionalized Au(III)‐alkyl complexes capable of undergoing photo‐induced β‐hydride elimination upon illumination at 420 nm in the presence of N‐acetylcysteine, releasing alkyl ligands and forming Au(III)‐hydride intermediates (**Figure** **7**A).^[^ ^118^ ^]^ These intermediates rapidly convert into biologically active [Au(III)–S] complexes and are subsequently reduced to highly cytotoxic Au(I) species, targeting thioredoxin reductase. The presence of glucose moieties facilitated tumor‐selective uptake, enhancing tumor specificity and therapeutic efficacy. This complex demonstrated significant inhibition of tumor angiogenesis in transgenic zebrafish embryos and robust antitumor activity in A375 tumor‐bearing mouse models.

In another example, Luo et al. (2020) designed cyclometalated Au(III)‐hydride complexes, wherein photoactivation at 420 nm led to hydride ligand dissociation, forming reactive [Au(III)‐S] complexes that specifically inhibited thioredoxin reductase. These complexes effectively induced apoptosis in HepG2 cancer cells and inhibited angiogenesis in zebrafish models.^[^ ^119^ ^]^ More recently, Xie et al. (2024) advanced this strategy by developing morpholine‐functionalized Au(III)‐hydride complexes.^[^ ^120^ ^]^ The morpholine moieties became positively charged in the acidic TME, forming ion pairs with negatively charged photocatalysts such as Eosin Y and Rose Bengal. This interaction resulted in a red‐shifted absorption spectrum in the visible and NIR region, facilitating activation by red light. Consequently, these complexes achieved tumor‐selective [Au(III)‐S] formation and effective thioredoxin reductase inhibition, overcoming the previously limiting ultraviolet activation dependence. Therapeutic efficacy was demonstrated against H3122 cancer cells and zebrafish tumor models.

### 6.2 Platinum‐Based Complexes (Pt(IV))

Pt(IV) complexes are particularly well‐suited to PACT due to their ability to undergo photoreduction, converting to highly cytotoxic Pt(II) species upon irradiation. Deng et al. (2020) developed Pt(IV) derivatives of oxaliplatin conjugated with coumarin‐based ligands at axial positions (Figure 7B).^[^ ^121^ ^]^ Upon irradiation at 450 nm, these complexes released coumarin ligands, leading to rapid reduction of Pt(IV) intermediates into active Pt(II) oxaliplatin species, inducing extensive DNA damage, p21‐mediated cell cycle arrest, apoptosis, and ICD. Enhanced cell penetration and nucleolar targeting were achieved through functionalization with the cell‐penetrating peptide R8K, significantly overcoming cisplatin resistance in lung cancer (A549cisR cells).

Further innovation by Deng et al. (2023) involved Pt(IV) oxaliplatin complexes capable of activation via two‐photon excitation under NIR (880 nm) irradiation.^[^ ^122^ ^]^ These complexes included coumarin‐based ligands designed for efficient two‐photon activation and dimethylxanthenone‐4‐acetic acid moieties for improved cellular uptake. Upon irradiation, photoreduction released active Pt(II) species and generated strong photooxidative stress, damaging intracellular membranes and disrupting pH homeostasis, ultimately inducing nonclassical necrosis and effectively overcoming drug resistance in lung cancer stem cells. In vivo studies showed significant inhibition of tumor growth, metastasis, and activation of antitumor immunity.

### 6.3 Ruthenium‐Based Complexes (Ru(II))

Ru(II)‐polypyridyl complexes undergo photosubstitution reactions involving solvent‐induced ligand dissociation, making them promising candidates for PACT.^[^ ^12^ ^]^ Havrylyuk et al. (2022) designed Ru(II) photocages conjugated to potent inhibitors of cytochrome P450 aromatase (CYP19A1), an enzyme critical for estrogen‐mediated tumor proliferation.^[^ ^123^ ^]^ Upon illumination at 660 nm, the inhibitor ligand was photoreleased and coordinated directly with CYP19A1's iron‐heme center, effectively suppressing its catalytic activity at picomolar concentrations. This system demonstrated remarkable enzyme inhibition efficacy in HEK293 T‐Rex cells, demonstrating the potential of Ru(II) complexes for highly selective targeted therapy.

## 7 Synergistic PAT: Metal‐Ligand Complexes for Enhanced PTT, PDT, and PACT

Recent advances in photo‐activated therapies have increasingly focused on harnessing the complementary mechanisms of PTT, PDT, and PACT to improve therapeutic efficacy. By combining multiple therapeutic modalities within a single integrated system, these synergistic strategies can effectively overcome limitations associated with individual treatments, such as tumor hypoxia, insufficient therapeutic selectivity, and acquired resistance mechanisms. To this end, metal‐ligand complexes have emerged as versatile platforms that can simultaneously serve as drug carriers, photosensitizers, photothermal agents, PACT drugs, and catalysts, all activated by a single light source. **Table** **5** presents representative recent examples of such multifunctional metal‐ligand complexes.

**Table 5:** Applications of metal‐ligand complexes for synergistic PAT: integrating PTT, PDT, PACT, and additional strategies for cancer treatment. This table presents therapy types, metal ions, ligands, roles in therapy, platform sizes, tumor models, targeted diseases, and light sources for activation.

Therapy type	Metal ion	Ligand	Role of metal‐ligand complex	Size	Tumor model	Disease	Light source	References
PTT, PDT	Ir(III)	1, 10‐phenanth‐roline‐5, 6‐dione, 2‐Phenylpyr‐idine, 2‐(2, 4‐Difluorop‐henyl)pyr‐idine	Photosensitizer, photothermal agent	‐	A375 tumor‐bearing mice	Cancer	808 nm	[126]
PTT, PDT	Ir(III)	Benzo[h]‐quinoline, N‐heterocyclic carbene	Photosensitizer	132.2 nm	4T1‐Luc tumor‐bearing mice	Cancer	660 nm	[127]
PDT, PACT	Au(III)	Bidentate C^N, alkyne	PDT‐to‐PACT drug	‐	Zebrafish, A375‐bearing mice, A375 cells	Cancer	420 nm, 630 nm	[128]
PDT, PACT	Ir(III)	2‐phenylpyr‐idine, monodent‐ate five‐membered heterocyclic ligands (imidazole, 1‐methyli‐midazole, or pyrazole)	Photosensitizer, PACT drug	‐	A549, A549R, PC3, and HepG2 cells	Cancer	425 nm	[129]
PDT, PACT	Ir(III)	2‐phenylpyr‐idine, 1, 10‐phenanth‐roline	Photosensitizer, PACT drug	116 nm	A549 tumor‐bearing mice, A549 cells (2D monolayer and multicellu‐lar tumor spheroids)	Cancer	750 nm	[130]
PDT, PACT	Ru(II)	2, 3‐bis(2‐pyridyl)‐benzo‐quinoxaline, pyridine	Photosensitizer	80 nm	HeLa and A549 cells	Cancer	427 nm	[131]
PDT, PACT	Ru(II)	Polypyridyl ligands, methionine and histidine residues	Photosensitizer, chemotherapy drug	100 nm	U87MG glioblast‐oma mice, A549, U87MG, and PC‐3 cells	Brain cancer	520 nm	[132]
PDT, PACT	Ru(II)	Bipyridine, 2, 9‐dimethyl‐1, 10‐phenanth‐roline	Photosensitizer	‐	4T1 cells, 4T1 tumor‐bearing mice	Cancer	700 nm	[133]
PTT, PDT, chemothe‐rapy	Zn(II)	Imidazole	Carrier	135 nm	MCF‐7 tumor‐bearing mice	Cancer	808 nm	[134]
PTT, PDT, CDT	Fe(III)	TA	Carrier, PTT agent	∼94 nm	MCF‐7 tumor‐bearing mice	Cancer	808 nm	[135]
PTT, PDT, CDT, chemothe‐rapy	Cu(II)	ICG	Carrier, Cu(II) as a cuproptosis agent, ICG as a photosensitizer	122.8 nm	H1975 tumor‐bearing mice, H1975 cells	Non‐small cell lung cancer	808 nm	[136]
PTT, PDT, CDT	Cu(II), Mn(II), Mn(III)	Tetraphe‐nylcarbox‐yporphyrin	Metal as a catalyst, ligand as a photosensitizer	71 nm	4T1 tumor‐bearing mice, MDA‐MB‐231 cells	Cancer	808 nm	[137]
PTT, PDT, immunoth‐erapy	Fe(III)	Pentagall‐oylglucose, Haase	Carrier, inhibitor of enzyme	255.3 nm of PPFH, 130.5 nm of IR780@LNPs	4T1, B16–F10 melanoma, and RM‐1 model in mice	Cancer	808 nm	[138]
PTT, PDT, chemothe‐rapy, immunoth‐erapy	Zn(II), Co(II)	Imidazole	Carrier, Co(II) for generation of oxygen	MOF of 150 nm, magnetic MOF of 180 nm	Tumor‐bearing mice	Cancer	808 nm	[139]
PDT, PACT, immunoth‐erapy	Ir(III), Fe(II)	Ferrocene‐tripyridine, phenyliso‐quinoline	Prodrug, Ir(III) as a photosensitizer, Fe(II) as a ferroptosis agent	87 nm	A375 cells, A375 cancer stem cells, B16–F10 tumor‐bearing mice	Melanoma	405 nm	[140]

### 7.1 Dual‐Modal PAT

#### 7.1.1 PTT/PDT Combinations

Conventional photosensitizers commonly used in PDT, such as [Ru(bpy)_3_]^2+^ and [Ir(ppy)_2_bpy]^2+^ (ppy = 2‐phenylpyridine) complexes, exhibit negligible photothermal conversion capability, limiting their use in combined PTT and PDT applications. To address this limitation, significant efforts have been made to design metal‐ligand complexes capable of simultaneously performing PTT and PDT.

**Figure 8:**
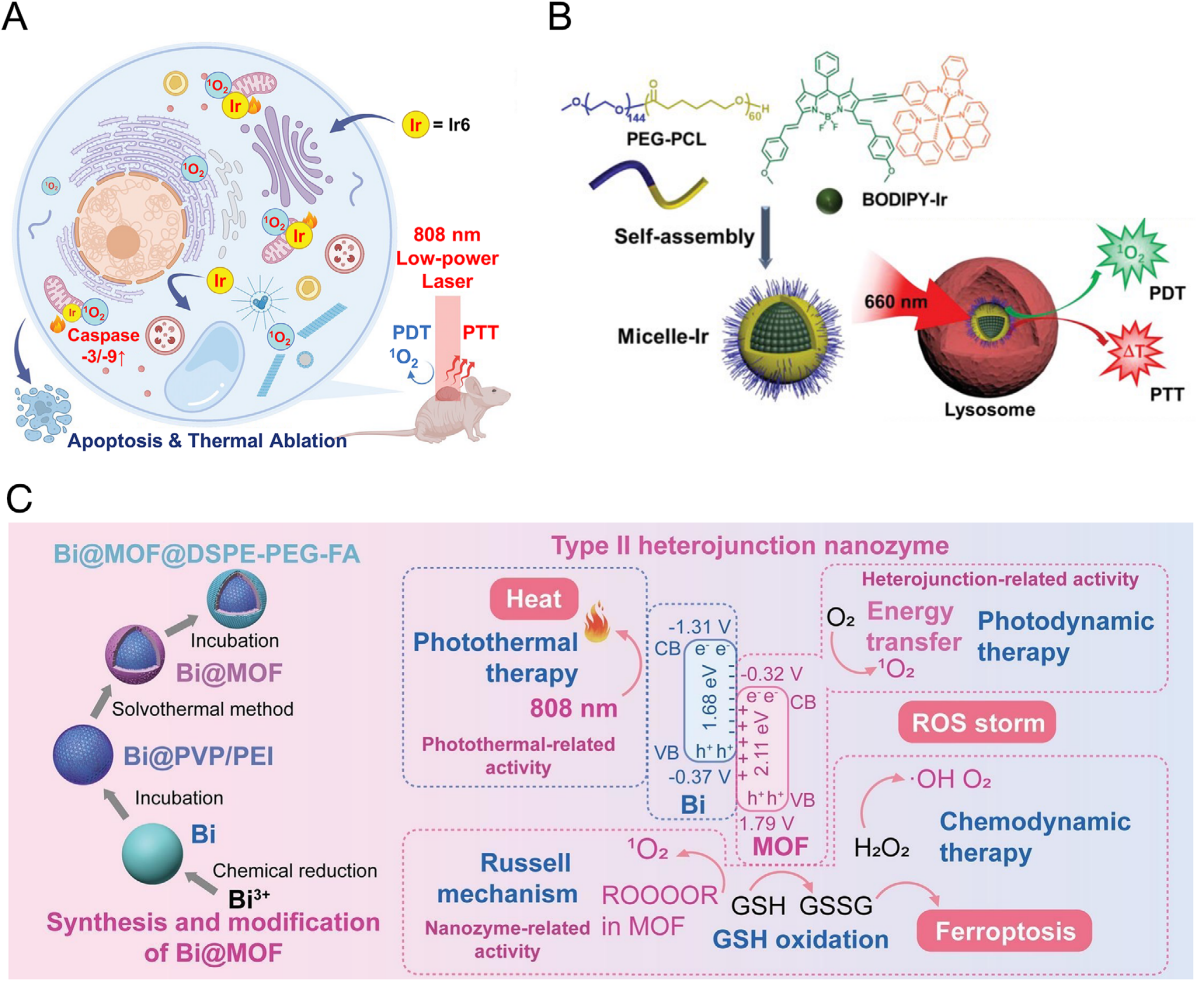
Representative studies employing metal‐ligand complexes for synergetic PAT. A) Schematic of the therapeutic mechanism of cyclometalated Ir(III) complexes as photosensitizers/photothermal agents. Reproduced with permission.^[^ ^126^ ^]^ Copyright 2023, Wiley. B) Illustration of the encapsulation of BODIPY‐Ir into micelles, forming Micelle‐Ir for intracellular PTT/PDT effects. Reproduced with permission.^[^ ^127^ ^]^ Copyright 2021, Wiley. C) Depiction of synthesis of Bi@MOF and Type II heterojunction‐based PTT/PDT effects. Reproduced with permission.^[^ ^137^ ^]^ Copyright 2025, Elsevier.

Tang et al. (2023) developed a series of Ir(III) cyclometalated complexes (Ir1–Ir6) incorporating morpholine and piperazine ligands (**Figure** **8**A).^[^ ^126^ ^]^ These complexes functioned as dual photosensitizers and photothermal agents, activated using low‐power infrared lasers for PAT. Although Ir1–Ir6 primarily absorb light in the 200–400 nm range, they can also undergo two‐photon absorption under 808 nm laser excitation, enabling their activation in the NIR region. The system demonstrated a maximum photothermal conversion efficiency of 18% and achieved a singlet oxygen quantum yield of ≈0.11. In vivo experiments using a dual PTT/PDT assay showed effective ablation of A375 tumors in mice under 808 nm laser irradiation.

Liu et al. (2021) developed an Ir(III)‐ligand complex capable of absorbing NIR light via one‐photon absorption, addressing limitations of existing Ir(III)‐ligand complexes that primarily rely on two‐photon absorption to operate in the NIR range.^[^ ^127^ ^]^ The earlier complexes suffered from low ROS generation efficiency and fast non‐radiative decay rates, reducing their therapeutic potential. The newly designed neutral Ir(III)‐ligand complex was based on distyryl BODIPY (BODIPY‐Ir), which effectively absorbed light in the 550–750 nm range, with a maximum absorption at 685 nm. The complex exhibited an extended triplet excited‐state lifetime, facilitating sufficient non‐radiative decay for photothermal and photodynamic effects. Through micellization, BODIPY‐Ir formed J‐type aggregates (Micelle‐Ir), which significantly enhanced ^1^O_2_ generation and photothermal efficiency (Figure 8B). The methoxy‐styryl units in the BODIPY core contributed electron‐donating properties and extended π‐conjugation, shifting the absorption spectrum into the far‐red/NIR regions (550–750 nm). Under 660 nm laser irradiation, the photothermal conversion efficiency of BODIPY‐Ir reached 28%, while micellized Micelle‐Ir achieved an improved efficiency of 30%. Furthermore, the micellization process resolved photobleaching issues, markedly enhancing the long‐term stability of the complex compared to non‐micellized BODIPY‐Ir. In vivo studies using a dual PTT/PDT assay demonstrated the therapeutic potential of Micelle‐Ir. When tested on 4T1 tumor‐bearing mice, PAT with 660 nm laser irradiation effectively eradicated tumors. The combined PTT and PDT effects, coupled with the extended stability and enhanced photothermal conversion efficiency, demonstrate the system's capacity to achieve robust tumor ablation while minimizing resistance due to single‐modality limitations.

#### 7.1.2 PDT/PACT Combinations

Metal‐ligand complexes have emerged as versatile agents capable of functioning both as photosensitizers for PDT and as prodrugs for PACT, leading to significant interest in their application as dual‐modal PDT/PACT cancer therapies. A key advantage of this strategy is the potential to activate distinct therapeutic pathways depending on local tumor conditions, such as oxygen availability. For instance, Luo et al. (2022) designed cyclometalated Au(III)‐alkyne complexes that display oxygen‐dependent photoactivation mechanisms, effectively acting as PDT‐to‐PACT agents.^[^ ^128^ ^]^ Under normoxic conditions, these complexes generate singlet oxygen catalytically upon visible‐light irradiation (420 nm), thus exhibiting potent PDT effects. However, under hypoxic conditions, excited states of the complexes are efficiently quenched by physiological thiols, triggering a photoreduction reaction that converts the complexes into cytotoxic Au(I) species, thus activating the PACT pathway. Furthermore, the photoreduction step can also be sensitized by external red‐light‐absorbing photosensitizers, such as riboflavin or protoporphyrin IX, enabling effective treatment at longer wavelengths (630 nm) and significantly expanding the therapeutic window beyond the conventional visible spectrum.

Wu et al. (2017) developed cyclometalated Ir(III) complexes based on 2‐phenylpyridine ligands incorporating monodentate five‐membered heterocyclic units, simultaneously performing PDT and PACT functions.^[^ ^129^ ^]^ Upon irradiation with 425 nm light, these complexes generated singlet oxygen and underwent dissociation of their monodentate ligands, releasing toxic species and inducing substantial cancer cell death. These complexes effectively increased intracellular ROS, reduced ATP concentrations, and induced apoptosis in cancer cells. Additionally, studies using human serum albumin as a model protein demonstrated that ligand dissociation under irradiation facilitated covalent binding of the metal centers to proteins, further confirming their potent phototoxic potential.

Kuang et al. (2022) designed mitochondria‐targeting cyclometalated Ir(III) endoperoxide complexes incorporating anthracene‐based endoperoxide ligands capable of functioning as reversible oxygen reservoirs.^[^ ^130^ ^]^ These complexes, composed of an Ir(III) center coordinated with 2‐phenylpyridine and anthracene‐functionalized 1, 10‐phenanthroline ligands, efficiently released cytotoxic Ir(III) species, singlet oxygen, and alkoxy radicals upon two‐photon NIR irradiation under hypoxic conditions. As a result, they exhibited nanomolar‐level cytotoxicity in hypoxic tumor cells and multicellular tumor spheroid models, accumulating preferentially in mitochondria and triggering the loss of mitochondrial membrane potential. Encapsulation within biotin‐functionalized amphiphilic polymers significantly improved tumor‐targeting specificity, pharmacokinetics, and therapeutic efficiency, achieving nearly complete tumor eradication in mouse models following just a single treatment.

Zhang et al. (2023) designed cyclic Ru‐peptide conjugates by coupling [Ru(Ph_2_phen)_2_(OH_2_)_2_]^2+^ (Ph_2_phen = 4,7‐diphenyl‐1,10‐phenanthroline) with an arginylglycylaspartic acid (RGD) tripeptide known for its high binding affinity to integrins overexpressed in tumor vasculature.^[^ ^132^ ^]^ The RGD‐containing peptide (Ac‐MRGDH‐NH2) included methionine and histidine residues, which formed coordination bonds with the Ru center. Under light irradiation, the peptide was released through photosubstitution, activating the Ru complex to penetrate cell membranes and induce cytotoxicity in cancer cells. This system demonstrated phototoxic effects in 2D A549, U87MG, and PC‐3 cell monolayers, as well as 3D U87MG tumor spheroids. Additionally, in a U87MG glioblastoma mouse model, the conjugates exhibited strong tumor‐targeting capabilities and significant antitumor efficacy through the combined action of PDT and PACT. This study demonstrates the potential of Ru‐based compounds for cancer therapy, offering improved tumor specificity and reduced side effects through controlled light‐activated mechanisms.

Zhu et al. (2024) developed a dual‐functional nanodrug by self‐assembling Ru(II) complexes with TH287, an inhibitor of MutT homolog 1 (MTH1), an enzyme involved in repairing oxidative DNA damage.^[^ ^131^ ^]^ The Ru(II) complexes generated singlet oxygen upon irradiation, concurrently releasing highly reactive Ru species that induced extensive DNA damage. TH287 synergistically inhibited MTH1‐mediated DNA repair pathways, thereby significantly enhancing overall cytotoxicity. The multifunctional nanodrug effectively induced ROS accumulation, oxidative DNA lesions, and apoptosis in tumor cells. Additionally, the Ru(II)‐derived ligand 2, 3‐bis(2‐pyridyl)benzoquinoxaline emitted red fluorescence upon release, enabling real‐time optical monitoring of drug uptake, intracellular distribution, and therapeutic efficacy.

To overcome the limited absorption of traditional Ru(II)‐based PACT complexes in the NIR region, Dao et al. (2024) synthesized a dinuclear Ru(II) complex featuring a donor–acceptor–donor (D–A–D) linker.^[^ ^133^ ^]^ This structural modification significantly prolonged the triplet excited‐state lifetime via intramolecular charge transfer processes, enabling efficient activation at 700 nm LED irradiation. Upon photoactivation, the complex generated singlet oxygen and exhibited catalytic photocatalytic activity, specifically oxidizing NADPH, a critical intracellular coenzyme involved in cellular redox balance. This redox disruption led to pronounced oxidative stress, endoplasmic reticulum stress, and excessive intracellular calcium influx, collectively triggering cell death pathways in 4T1 cancer cells at nanomolar concentrations. In vivo, the complex displayed significant antitumor effects in 4T1 tumor‐bearing mouse models, demonstrating its potential for highly effective PDT/PACT dual therapy.

### 7.2 Multifunctional Platforms for Combined Therapies

Chen et al. (2023) reported a system combining PTT, PDT, and chemotherapy using DOX/ICG‐loaded ZIF‐8 nanoparticles (ZS/ID NPs).^[^ ^134^ ^]^ These nanoparticles were synthesized using silk fibroin as a template, along with Zn^2+^ and 2‐methylimidazole to form the ZIF‐8 framework. After loading DOX and ICG into the porous ZIF‐8 structure, the nanoparticles were coated with polyethylenimine for enhanced stability and surface modification with the breast tumor‐targeting peptide AREYGTRFSLIGGYR (AR peptide). The AR peptide facilitated selective accumulation of the nanoparticles in MCF‐7 breast tumors, utilizing the peptide's affinity for tumor‐specific markers. Once localized in the tumor, the acidic microenvironment triggered the degradation of the ZIF‐8 framework, releasing DOX and ICG. DOX functioned as a chemotherapeutic agent, while ICG acted as a photosensitizer and photothermal agent. Upon 808 nm laser irradiation, ICG generated ROS for PDT and heat for PTT, synergistically promoting cancer cell apoptosis. In vivo studies in an MCF‐7 tumor‐bearing mouse model demonstrated significant tumor inhibition. The combined effects of chemotherapy, PDT, and PTT led to improved therapeutic efficacy compared to single‐modality treatments. The acidic pH‐responsive release of DOX and ICG further enhanced the system's efficiency by ensuring controlled drug delivery and minimizing off‐target effects. The multifunctional ZS/ID NPs illustrate the potential of integrating photothermal and photodynamic therapies with conventional chemotherapy to achieve synergistic cancer treatment, addressing limitations of individual modalities. Li et al. developed a nanoplatform (UCNP@HHS@Fe‐TA) combining UCNPs with hydrophobic hydration shells (HHS) containing perfluorocarbons (PFCs) and Ce6, and coated with Fe‐TA for enhanced CDT.^[^ ^135^ ^]^ The PFCs improved oxygen delivery, while Ce6 generated ROS under NIR irradiation through FRET. Simultaneously, Fe‐TA contributed to PTT and accelerated hydroxyl radicals generation via Fenton reactions, achieving enhanced anti‐tumor effects in MCF‐7 models.

Liu et al. (2025) recently developed a multifunctional nanoparticle system through metal‐ligand coordination self‐assembly, targeting non‐small cell lung cancer. This nanoplatform incorporates the epidermal growth factor receptor (EGFR) tyrosine kinase inhibitor osimertinib, Cu^2+^, and the photosensitizer ICG.^[^ ^136^ ^]^ Osimertinib served as a chemotherapy agent, inhibiting EGFR‐mediated tumor proliferation, and delivered Cu^2+^ ions selectively targeted lipoylated proteins within the cellular tricarboxylic acid cycle, inducing protein aggregation and causing the loss of iron–sulfur (Fe–S) cluster proteins. This disruption elevated proteotoxic stress, triggering cell death through cuproptosis. Additionally, Cu^2+^ participated in CDT by generating ROS through Fenton‐like reactions with hydrogen peroxide in the TME. The generated ROS further enhanced oxidative stress by promoting lipid peroxidation and GSH depletion, thereby sensitizing cancer cells. Under NIR (808 nm) irradiation, ICG significantly enhanced PDT efficacy by further amplifying ROS production. Moreover, ICG also mediated photothermal effects, locally elevating tumor temperature and potentiating the cytotoxic effects of Cu^2^⁺‐induced Fenton reactions and osimertinib‐driven chemotherapy. Consequently, this multifunctional nanoparticle demonstrated excellent therapeutic efficacy against H1975 cells and corresponding mouse tumor models by synergistically integrating cuproptosis, chemotherapy, CDT, PDT, and PTT into a single, potent cancer treatment strategy.

Similarly, Zhang et al. (2025) designed a core‐shell plasmonic heterojunction nanoparticle platform comprising bismuth nanoparticles (Bi NPs) at the core and porphyrin‐based MOFs containing Cu and Mn ions at the shell (Figure 8C).^[^ ^137^ ^]^ To improve colloidal stability, systemic circulation, and tumor‐targeting capability, nanoparticles were further surface‐functionalized with DSPE‐PEG‐folic acid. Upon irradiation at 808 nm, Bi NPs induced significant photothermal effects via surface plasmon resonance, simultaneously generating energetic excitons. The plasmon‐induced excitons facilitated electron transfer to the MOF shell, efficiently converting molecular oxygen into cytotoxic ROS, thus enabling effective PDT. Additionally, Cu and Mn ions within the MOF shell participated in CDT through Fenton‐like reactions, catalyzing ROS production from hydrogen peroxide abundant in the TME. The oxidation reactions further led to significant intracellular GSH depletion, triggering ferroptosis. Moreover, Cu and Mn ions interacted with peroxyl radicals to enhance singlet oxygen production, synergistically amplifying CDT efficacy. Collectively, upon NIR irradiation, these integrated mechanisms resulted in substantial anticancer effects against MDA‐MB‐231 breast cancer cells and 4T1 tumor‐bearing mice, effectively combining PTT, PDT, CDT, and ferroptosis into a single therapeutic system.

### 7.3 Combined Tumor Therapy and Immune Modulation

Sun et al. (2024) reported a multifunctional system (PPFH) combining PTT and PDT for the treatment of solid tumors and the induction of immune responses.^[^ ^138^ ^]^ The PPFH system was constructed using a Fe^3+^‐based MPN, formed through coordination between Fe^3+^ ions and pentagalloylglucose, with hyaluronidase (HAase) encapsulated to create nanoparticles. In the nanoparticle state, Fe^3+^ ions bind to the active sites of HAase, suppressing its enzymatic activity and preventing premature activity before reaching the tumor site. Following nanoparticle injection, the iron‐chelating agent deferoxamine (DFO) was administered to selectively chelate Fe^3+^ ions, disassembling the MPN structure and releasing HAase within the tumor. Once released, HAase acted as a hydrolytic enzyme, breaking down the dense tumoral extracellular matrix (TECM) and reducing physical barriers to therapeutic penetration. This TECM‐modulating process was complemented by the introduction of liposomes loaded with the NIR photosensitizer IR780 iodide (IR780@LNPs) for PTT and PDT. These liposomes penetrated the loosened TECM and accumulated within the tumor. Upon irradiation with an 808 nm laser, the system achieved enhanced PTT and PDT efficiency while inducing ICD. The combined effects of PTT and PDT caused tumor damage, which was amplified by the TECM degradation facilitated by activated HAase. This allowed activated CD8^+^ T cells to infiltrate the tumor, while tumor antigens released from the damaged tissue were efficiently captured by DCs, boosting the immune response. The combination of PPFH, DFO, PTT, and PDT demonstrated robust therapeutic effects in 4T1, B16–F10, and RM‐1 tumor‐bearing mouse models. The system effectively suppressed tumor growth, prevented recurrence and metastasis, and significantly prolonged survival times.

Bai et al. (2024) developed a multifunctional Zn/Co‐MOF system to enhance tumor immunotherapy through the combination of PTT and PDT.^[^ ^139^ ^]^ The system consisted of two types of Zn/Co‐MOFs: one loaded with metformin, cisplatin, and glucose oxidase (GOD), and the other magnetic Zn/Co‐MOF loaded with 1‐methyl‐tryptophan (1‐MT), cisplatin, and ICG. Both types of MOFs were coated with polydopamine to improve biocompatibility, and their surfaces were further functionalized with niobium carbide (Nb_2_C) Mxene quantum dots (QDs) and AS1411 aptamers for tumor targeting. The nanoreactor achieved selective tumor accumulation through the synergistic effects of the aptamer and a magnetic field. Once localized, the acidic TME triggered the degradation of polydopamine, releasing metformin and 1‐MT. Within the TME, the GOD‐mediated reaction consumed endogenous glucose to generate hydrogen peroxide and gluconic acid. The produced hydrogen peroxide was subsequently converted into oxygen by Co^2+^ ions, while ROS generation by ICG improved the hypoxic environment, reducing PDT resistance. Simultaneously, the photothermal properties of Nb_2_C QDs enabled localized heating upon 808 nm laser irradiation, inducing tumor cell death and releasing tumor‐associated antigens within the tumor. Metformin inhibited hexokinase 2 activity, blocking glycolytic pathways and reducing ATP production, which effectively suppressed tumor cell proliferation. Furthermore, metformin reversed tumor immune suppression by promoting the cytoplasmic degradation of PD‐L1. The released 1‐MT regulated the immunosuppressive TME, enhancing the immune response. The combined use of metformin and 1‐MT stimulated antitumor immunity, as demonstrated in the 4T1 tumor‐bearing mouse model, where the system effectively inhibited tumor growth and triggered a robust immune response. This multifunctional nanoplatform demonstrates the potential to integrate PTT, PDT, and immunotherapy to address the limitations of single‐modality treatments, improving therapeutic outcomes while simultaneously targeting both cancer cells and the immunosuppressive TME.

More recently, Lie et al. (2025) reported a photo‐activated ferrocene‐Ir(III) complex exhibiting potent antitumor effects through the integrated mechanisms of PDT, PACT, ferroptosis, and immunotherapy.^[^ ^140^ ^]^ Specifically designed to target mitochondria, the complex undergoes photo‐induced decomposition upon irradiation at 405 nm, releasing cytotoxic Ir(III) species and Fe^2+^ ions. The Ir(III) complex generates ROS through efficient photosensitization, while simultaneously, the released Fe^2+^ ions induce additional ROS production via the Fenton reaction. This combined ROS generation triggers ferroptosis and autophagy, subsequently promoting the release of DAMPs, which ultimately leads to ICD. Moreover, this multifunctional complex effectively suppresses stemness‐associated markers within tumor tissues, significantly reducing melanoma cell self‐renewal capabilities. It also enhances the phagocytosis of melanoma stem cells by TAMs, further potentiating its antitumor immune response. To optimize tumor specificity and therapeutic efficacy, the ferrocene‐Ir(III) complex was encapsulated within tumor‐targeting nanoparticles composed of DSPE‐PEG. In a mouse melanoma (B16–F10) model, these nanoparticles exhibited excellent antitumor efficacy upon photoactivation, effectively eliminating primary and distant tumors, including melanoma stem cells, and demonstrating substantial inhibition of tumor metastasis and recurrence. Taken together, these findings demonstrate the remarkable potential of integrated PDT/PACT strategies using metal‐ligand complexes, illustrating their capability to simultaneously induce direct cytotoxic effects, promote ferroptosis, and activate systemic antitumor immunity, thereby significantly improving antitumor therapy.

## 8 Conclusions and Future Perspectives

This review explores the unique optical properties and tunable characteristics of metal‐ligand complexes in PAT. These features facilitate efficient ROS generation and energy or charge transfer via light absorption for precise cancer cell targeting and destruction. We classified metal‐ligand complexes based on their ligands, such as polyphenols, MOF ligands, and tetrapyrrolic ligands, as well as the metal ions they incorporate, including transition and noble metals, highlighting their roles in improving therapeutic efficacy. Recent developments in combined therapies, such as chemo‐phototherapy and immunotherapy, were also discussed, illustrating their potential for synergistic tumor treatment. Despite significant progress in metal‐ligand complex‐based phototherapies, several critical aspects require further investigation to maximize their therapeutic potential. Here, we discuss key perspectives toward the rational design of next‐generation metal‐ligand complexes that offer precise, effective, and clinically translatable cancer phototherapies.

### 8.1 Unraveling the Multifunctionality of Metal‐Ligand Complexes: A Mechanistic Perspective

Metal‐ligand complexes have been extensively explored for their diverse roles in PAT, serving as photothermal agents, photosensitizers, and carriers for therapeutic agents. However, most studies have lacked a thorough mechanistic elucidation of their therapeutic effects, as PTT, PDT, and CDT often coexist in therapeutic applications.

Current explanations often treat PDT and CDT (especially using the Fenton reaction or Fenton‐like reaction) as distinct processes. PDT relies on light‐activated photosensitizers to generate ROS, while the Fenton reaction or Fenton‐like reaction is attributed to Fe^2+^‐mediated hydroxyl radicals formation from hydrogen peroxide. Many studies indicate that heat from photothermal agents can enhance the efficiency of the Fenton reaction. In this context, iron ions released from metal‐ligand complexes typically function as catalysts for this reaction. However, this explanation may be oversimplified and overlook the potential photochemical role of metal‐ligand complexes. Instead, a more comprehensive view considers metal‐ligand complexes as photoactive entities that absorb light, facilitate Fe redox cycling, and enhance ROS production without requiring Fe ion release.

For example, MPNs formed by Fe ions and polyphenols can absorb NIR light and be utilized for PTT, while released Fe ions contribute to CDT through the Fenton reaction. However, MPNs have not yet been explored as direct photosensitizers for PDT. In contrast, research in the field of photocatalysis has demonstrated that metal‐ligand complexes can undergo charge transfer excitations, altering the oxidation state of Fe ions and generating ROS through Fenton‐like reactions.^[^ ^141^, ^142^ ^]^ Furthermore, polyphenols can oxidize into quinones under light irradiation, facilitating Fe redox cycling and enhancing the Fenton reaction.^[^ ^143^ ^]^ These findings suggest that PDT and Fenton reaction (or Fenton‐like reaction) could be more closely linked than previously thought, with light‐induced Fe oxidation‐state modulation playing a crucial role in ROS production. Consequently, leveraging insights from photocatalysis could lead to innovative approaches for metal‐ligand complex‐based treatments. Specifically, in Fe‐ligand systems, LMCT could drive Fe oxidation‐state changes, triggering ROS production for PDT and CDT without requiring Fe ion release. At the same time, LMCT could enhance NIR absorption, facilitating photothermal conversion for PTT while accelerating ROS generation. This concept could be extended to other metal‐ligand systems, broadening their therapeutic potential.

Future research should extend beyond simply demonstrating therapeutic efficacy. Instead, a systematic investigation into the fundamental photophysical and photochemical interactions underlying metal‐ligand complexes is necessary to fully realize their potential in PAT. Time‐resolved spectroscopy, in situ monitoring, and computational modeling could provide critical insights into how metal‐ligand complexes function as both photosensitizers and redox catalysts. By clearly delineating the interplay between PTT, PDT, CDT, and ferroptosis, researchers can design metal‐ligand complexes with tailored functionalities, optimizing treatment strategies for cancer therapy. In conclusion, metal‐ligand complexes hold significant promise for multifunctional PAT, but their full potential remains untapped. By integrating concepts from photocatalysis and refining our understanding of their photochemical mechanisms, future research can pave the way for more effective and synergistic cancer treatments.

### 8.2 Enhancing the Safety and Efficacy of Metal‐Ligand Complexes

The incorporation of metal‐ligand complexes into nanostructures such as MOFs or MPNs provides additional opportunities to optimize their safety and efficacy. MOFs and MPNs can act as carriers for metal ions, shielding them from premature release and ensuring stability during circulation. These nanostructures can also be functionalized with targeting moieties such as peptides, antibodies, or aptamers to enhance tumor specificity. Furthermore, their porous structures enable the co‐delivery of multiple therapeutic agents, such as photosensitizers or chemotherapeutics, facilitating combination therapies.

While metal‐ligand complexes have demonstrated considerable potential, further progress in their design is required to address challenges such as systemic toxicity, off‐target effects, and limited tumor specificity. Integrating advanced imaging techniques with therapeutic functions, such as theranostics, can provide real‐time feedback on the biodistribution and therapeutic effects of these complexes. Moreover, exploring novel ligand chemistries and hybrid nanostructures may yield systems with enhanced selectivity and efficacy, paving the way for their widespread clinical translation.

## Conflict of Interest

The authors declare no conflict of interest.
